# Monocyte infiltration and proliferation reestablish myeloid cell homeostasis in the mouse retina following retinal pigment epithelial cell injury

**DOI:** 10.1038/s41598-017-08702-7

**Published:** 2017-08-16

**Authors:** Wenxin Ma, Yikui Zhang, Chun Gao, Robert N. Fariss, Johnny Tam, Wai T. Wong

**Affiliations:** 1 Unit on Neuron-Glia Interactions in Retinal Disease, National Institutes of Health, Bethesda, MD 20892 USA; 2 Biological Imaging Core, National Eye Institute, National Institutes of Health, Bethesda, MD 20892 USA; 3 Ophthalmic Genetics and Visual Function Branch, National Eye Institute, National Institutes of Health, Bethesda, MD 20892 USA

## Abstract

Age-related macular degeneration (AMD), a leading contributor of vision loss, currently lacks comprehensive treatment. While AMD histopathology involves retinal pigment epithelium (RPE) injury associated with immune cell infiltration, the nature of immune cell responses to RPE injury remains undefined. We induced RPE injury pharmacologically and genetically in transgenic mouse models in which microglia and systemic monocytes were separately tagged, enabling a spatial and temporal dissection of the relative contributions of microglia vs. monocytes to post-injury changes. We found that myeloid cell responses to RPE injury occur in stages: (1) an early mobilization of endogenous microglia from the inner retina to the RPE layer, followed by (2) subsequent monocyte infiltration from the retinal vasculature into the inner retina that replenishes the local myeloid cell population in a CCR2-regulated manner. These altered distributions of myeloid cells post-injury were long-lived, with recruited monocytes acquiring the distribution, markers, and morphologies of neighboring endogenous microglia in a durable manner. These findings indicate the role played by infiltrating monocytes in maintaining myeloid cell homeostasis in the retina following AMD-relevant RPE injury and provide a foundation for understanding and therapeutically modulating immune aspects in retinal disease.

## Introduction

Microglia in the central nervous system (CNS) constitute a stable resident population of innate immune cells that are constitutively required to maintain proper synaptic function subserving learning and cognition^1, 2^. In the retina, microglia in the adult animal have been shown to be required for maintaining healthy synaptic structure and function subserving normal vision^3^. Retinal microglia demonstrate a tiled and regular spatial distribution in the inner retina and participate in dynamic contact with retinal neurons and macroglia via motile, ramified processes^4^, indicating their active role in communication with other retinal cells^5, 6^. Conversely, retinal microglia in pathological situations have been thought to contribute to disease pathogenesis and progression of retinal diseases; in these situations, microglia transition to an activated phenotype, migrate to areas of pathology, and potentiate cellular degeneration in disease lesions^7–9^. Although microglia in the CNS represent a closed population of self-sustaining cells under normal conditions^10^, infiltration of systemic monocytes can occur in disease, contributing an additional population of myeloid cells to the overall CNS milieu^11^. As markers that distinguish between endogenous microglia and exogenous monocyte-derived cells are not yet well developed, the relative involvement and contribution of these myeloid cells to pathological vs. adaptive responses are not clearly defined^12^. In the retina, these uncertainties have complicated the elucidation of mechanisms underlying retinal diseases involving immune cells and have limited the formulation of immunomodulatory therapeutic strategies^13^.

Age-related macular degeneration (AMD), a major significant cause of blindness in the developed world, is a retinal disease in which photoreceptor and retinal pigment epithelium (RPE) degeneration contribute to vision loss. The inflammatory etiology of AMD has been strongly indicated by genome-wide association studies (GWAS) associating inflammatory genes with AMD risk^14^, and have been supported by studies localizing immune myeloid cells to disease lesions on histopathology in AMD human specimens^15–18^ and mouse models of AMD^19^. The detection of innate immune cells at the retinal pigment epithelium (RPE)-Bruch’s membrane complex has prompted the hypothesis that interactions between immune cells and the RPE are influential in the pathobiology of AMD^20, 21^. However how RPE injury in AMD may induce changes in the number, composition, and distribution of resident myeloid cell populations in the retina is unclear, as is the systemic vs. endogenous sources for these myeloid cells that aggregate at sites of RPE injury. Knowledge as to how myeloid cells in the retina respond to RPE changes, and which populations of myeloid cells participate in reactive vs. adaptive responses will help provide a foundation for the discovery of pathogenic immune mechanisms^22, 23^.

In the current study, we examined the dynamic responses of myeloid cells in the retina to RPE injury using pharmacological and genetic models that induce RPE cell death in experimental mice. We employed a genetic method of “cell fate-mapping” to differentially label endogenous retinal microglia vs. exogenous infiltrating monocytes in our experiments in order that cellular responses to RPE injury, such as infiltration, migration, proliferation, and changes in morphology, can be tracked separately in each myeloid cell population. In addition, we obtained corroborative data of monocyte infiltration dynamics using CCR2^RFP/+^ transgenic mice in which CCR2-expressing monocytes are labeled with red fluorescent protein (RFP). This transgenic system also enabled the contribution of CCR2-mediated signaling in RPE injury-induced responses to be examined. We discovered in this study that RPE injury induced a rapid mobilization of myeloid cells to the subretinal space that were constituted primarily by endogenous microglia recruited from the inner retina with little contribution from systemic monocytes. Interestingly, this early injury response was coordinated with a subsequent homeostatic response in which proliferating systemic monocytes infiltrated into the inner retina via the retinal vasculature to replace the microglia that had migrated to the subretinal space. These monocytes infiltrated the retina in a CCR2-regulated manner, established residence in the plexiform layers, and developed ramified morphologies similar to those found in endogenous microglia. These observations indicated that innate immune cell responses may be coordinated between myeloid cells that are directly responsive to injury and are directed to injury sites with those that serve to maintain myeloid cell homeostasis in the aftermath of the injury response. Taken together, we discovered that overall myeloid cell responses in retinal injury involve both endogenous microglia and exogenous monocytes, with each population contributing to different phases of the overall response in a coordinated manner. The discovery of the complexities of myeloid cell dynamics enabled by tracking of separate myeloid cell populations can provide insight  into immune mechanisms in retinal disease such as AMD.

## Materials and Methods

### Experimental Animals

CX3CR1^GFP/+^ mice were produced by crossing CX3CR1^GFP/GFP^ ^24^ and wild type C57BL/6 J mice, CX3CR1^GFP/+^:CCR2^RFP/+^ mice by crossing CX3CR1^GFP/GFP^ and CCR2^RFP/RFP^ mice^25^, CX3CR1^CreER/+^: Rosa26-flox- STOP-flox-tdTomato (hereafter termed Cre-tdT) mice by crossing homozygous CX3CR1^CreER^ ^1^ (courtesy of Dr. Wenbiao Gan) and Rosa26-flox-STOP-flox-tdTomato^26^ mice. VMD2-Cre; iDTR; CCR2^RFP/+^ mice were produced by interbreeding VMD2-Cre (Cat. 017557)^27^, CCR2^RFP/RFP^ mice, and ROSA26iDTR mice (Cat. #007900) mice. VMD2-Cre; Rosa26-iZSGreen mice were produced by interbreeding VMD2-Cre mice and ROSA26iZSGreen mice (Cat. #007906). All mouse strains were obtained from The Jackson Laboratory unless otherwise specified. All experimental animals were genotyped by gene sequencing to confirm the absence of the rd8 mutation before further experimentation^28^. To induce Cre-ERT-mediated recombination and expression of tdTomato in CX3CR1-expressing cells, two-month old Cre-tdT mice were administered tamoxifen dissolved in corn oil (Sigma-Aldrich; 500 mg/kg dose of a 20 mg/ml solution) via oral gavage (two doses, one day apart). These animals were kept under standard vivarium conditions and used in subsequent experiments 3 months later. To label proliferating cells, BrdU (Sigma-Aldrich; 50 μg/g body weight) was injected intraperitoneally into experimental animals daily for 3 consecutive days before analysis. All animals were bred and housed in a National Institutes of Health animal facility under a 12-h light/dark cycle with food *ad libitum*. Experiments were conducted consistent with protocols approved by the National Eye Institute Animal Care and Use Committee and adhered to the ARVO Statement for the Use of Animals in Ophthalmic and Vision Research.

### Sodium iodate (NaIO_3_)-induced and diphtheria toxin-induced models of RPE Cell Injury

In the NaIO3-induced model of RPE injury, two month-old CX3CR1^GFP/+^, CX3CR1^GFP/+^: CCR2^RFP/+^, and CCR2^RFP/RFP^ mice and 5-month old Cre-tdT mice (previously administered oral tamoxifen at 2 months of age) were administered a single dose of NaIO_3_ (Honeywell Research Chemicals:, 30 mg/kg body weight) via intraperitoneal injection. Animals were euthanized and their retinas analyzed 1 to 180 days following NaIO_3_ administration. In the diphtheria toxin-induced model of RPE injury, diphtheria toxin (DTA) (Sigma) was administered to VMD2-Cre, iDTR; CCR2^RFP/+^ mice via intraperitoneal injection (two doses over 3 days, 1 µg dissolved in PBS per dose). Treated animals were euthanized and retinal tissue was harvested for analysis at 3d and 7d following the final DTA dose.

### Immunohistochemistry

Eyecups were fixed with 4% paraformaldehyde for 2 h before transfer into PBS. Fixed eyecups were either sectioned into 16μm-thick sections with a cryostat or dissected to generate retinal and sclerochoroidal-RPE flatmounts. Tissue samples were blocked for 1 h in blocking buffer containing 6% normal goat serum and 1% Triton X-100 in PBS at room temperature. Primary antibodies against the following antigens were used: Iba1 (Wako, 1:500), Ki67 (Abcam, 1:50), nestin (Santa Cruz, 1:300), BrdU (Developmental Studies Hybridoma Bank, 1:100), and ZO-1 (Invitrogen, 1:200), CCL2 (R&D, AB-479, 1:100), CCL7 (R&D, AF-456, 1:100), I-A/I-E (BD Biosciences, 1:40), CD45 (AbD Serotec, 1:100), and F4/80 (AbD Serotec, 1:100). Secondary antibodies that were raised in goat, donkey, or chick and conjugated to either Alexa Fluor-488, −568, or −633 (Invitrogen, 1:200) were used. Phalloidin-conjugated to AlexaFluor-633 (Invitrogen, 1:200), and isolectin-B4 (IB4) lectin (Life Science, 1:200) were used as probes to F-actin and terminal a-D-galactosyl residues on vascular endothelial cells respectively. Primary antibodies to CCL2 and CCL7 were evaluated by Western blotting, and were each confirmed to generate a positive band of the expected size.

Spleens were dissected from Cre-tdT animals 2 weeks and 3 months following tamoxifen administration, fixed in 4% paraformaldehyde, and sectioned into 16μm-thick sections with a cryostat. Spleen macrophages in the sections were immunolabeled for Iba1 (Wako,1:500) as above. Blood samples (0.5 ml) were concurrently obtained from these animals and incubated in ACK lysis buffer (Lonza, Walkersville) for 10 min to lyse red blood cells. Remaining leukocytes were centrifuged, washed, and labeled with a primary antibody to CD11b conjugated with Fluor488 (1:20, eBioscience, San Diego) in staining buffer (BD Pharmingen, San Diego) for 20 min on ice. Labelled cells were washed, smeared on a glass slide, fixed briefly in 2% paraformaldehyde for 10 minutes, and allowed to dry, before mounting in a medium containing DAPI (Vectashield, H-1200, Vector Laboratories, Burlingame).

### Measurement of cytokine levels in retinal tissue

Dissected mouse retinas were lysed by trituration in protein lysate buffer (Complete Ultra; Roche) with proteinase inhibitor mixture (Calbiochem) at 4 °C. After sonication and centrifugation, protein concentration was measured (BCA protein assay kit; Pierce). Cytokine levels were determined using a Milliplex bead assay kit (Milliplex MAP mouse cytokine/chemokine magnetic bead panel, #MCYTOMAG-70K; Millipore) using the Luminex MAPIX system with data analysis using xPONENT 4.2 software (Luminex).

### Image capture and analysis

Labelled samples were imaged with confocal microscopy (Olympus FV-1000 and Zeiss 880 confocal microscopes). Cell counts in separate retinal lamina were made from 20x and 40x confocal images taken in stained retinal and sclerochoroidal-RPE flatmounts using computer-assisted methods (Image J software, NIH). Regions of interest for cell counting were identified around the optic nerve and in the mid-peripheral retina at distances between 0.75 and 1.25 mm from the optic nerve. Image analyses were drawn from groups consisting of 3-6 biological repeats in each group. Three-dimensional morphological analyses of microglia were performed using computer-assisted segmentation of microglial processes (Filament Tracer module and the Convex Hull Xtension feature, Imaris software). Morphological parameters quantitated were: (1) total dendritic process length in a microglial cell, (2) total number of dendrite segments per cell, (3) total number of branching points in a microglial dendritic field, and (4) mean volume occupied per cell (the 3-dimensional volume subtended by a single microglial dendritic field computed as the volume of the convex hull enclosing the dendritic arbor).

### mRNA analysis by quantitative PCR

Expression of mRNA was quantitated using quantitative reverse transcription-PCR (qRT-PCR). Retinas were harvested from CX3CR1^GFP/+^: CCR2^RFP/+^ mice at 0d, 3d, 7d and 30d following NaIO_3_-induced injury and VMD2:iDTR:CCR2 mice at 0d, 3d following DTA last injection. Cells in retina tissue were lysed by trituration and homogenized using QIAshredder spin columns (Qiagen). Total RNA was isolated using the RNeasy Mini kit (Qiagen) according to the manufacturer’s specifications. First-strand cDNA synthesis from mRNA was performed using qScript cDNA SuperMix (Quanta Biosciences) using oligo-dT as primer. qRT-PCR was performed using a SYBR green RT-PCR kit (Affymetrix), using the Bio-Rad CFX96 Touch™ Real-Time PCR Detection System under the following conditions: denaturation at 95 °C for 5 min, followed by 40 cycles of 95 °C for 10 s, and then 60 °C for 45 s. Threshold cycle (CT) values were calculated, and expressed as fold-induction determined using the comparative CT (2ΔΔCT) method. Ribosomal protein S13 (RPS13) and GAPDH were used as internal controls. Oligonucleotides primers used are provided in Supplementary Table 1.

### Statistical Analysis

All data were analyzed using statistical software (GraphPad Prism Software, Version 6.0.1). A normality test (D’Agostino and Pearson) was used to analyze the distribution of all data sets. For two-way comparisons of data following a Gaussian distribution, independent data sets were analyzed with an unpaired two-tailed t-test; those not following a Gaussian distribution were evaluated with a Mann-Whitney test. For comparisons of data in three or more groups, a one-way ANOVA was used. A P-value < 0.05 was set as the basis for rejecting the null hypothesis. For comparisons determining the effect of genotype (CCR2^RFP/+^ vs. CCR2^RFP/RFP^ mice) at different times following NaIO_3_ administration, a two-way ANOVA was employed. In all graphical representations, the error bars indicate standard error (SE).

## Results

### Dynamic changes in the distribution patterns of myeloid cells in the retina following NaIO_3_-induced RPE injury

**Figure 1:**
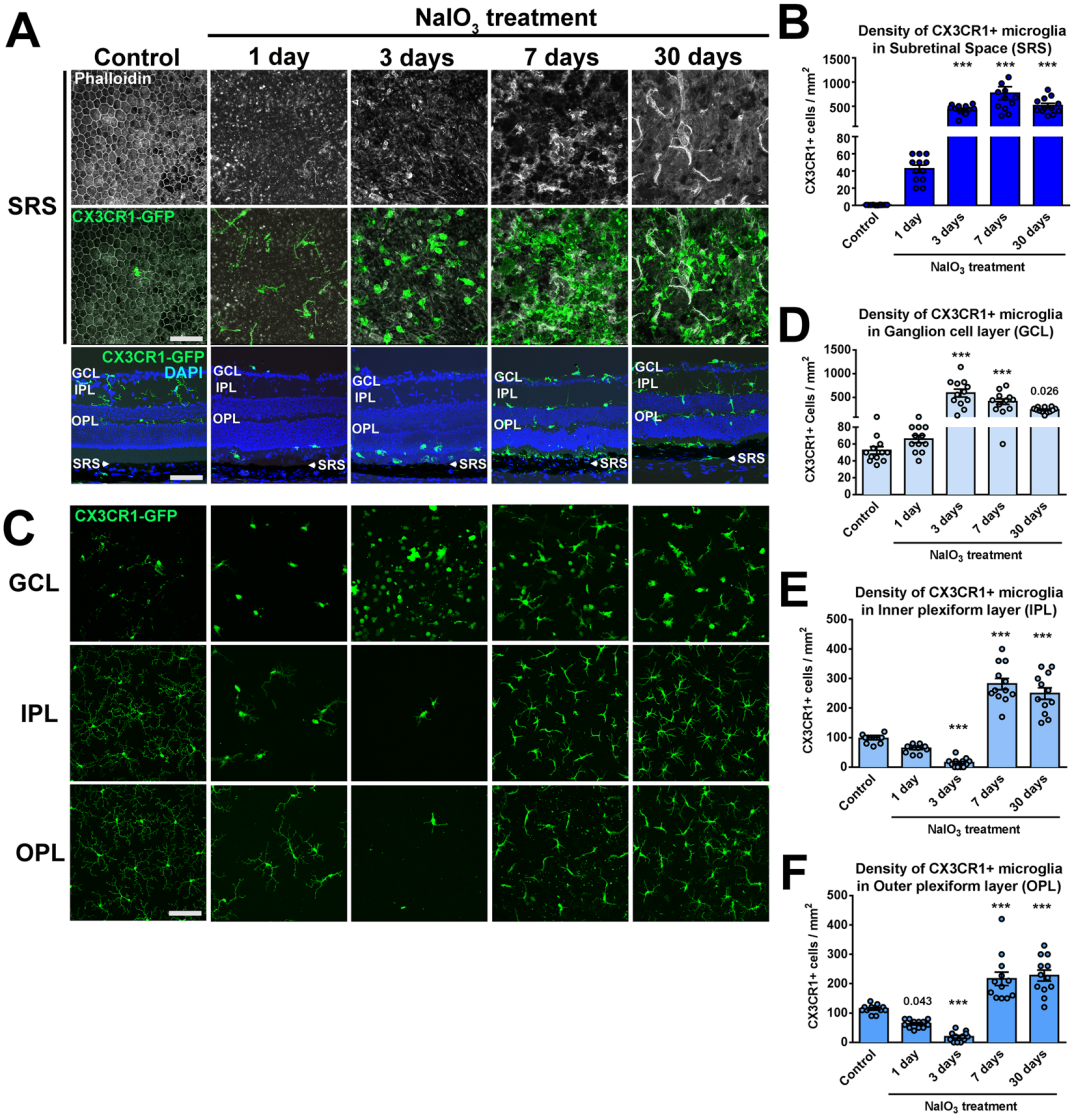
NaIO_3_-induced RPE injury induces time-dependent changes in the distribution of CX3CR1-expressing myeloid cells in inner vs. outer retinal lamina. (**A**) Immunohistochemical analysis of sclerochoroidal flat-mounts from 2-month old CX3CR1^GFP/+^ mice at 1, 3, 7 and 30 days following a single dose of NaIO_3_ administration (delivered by intraperitoneal injection, 30 mg/kg dose) demonstrated rapid-onset disorganization and degeneration of phalloidin-labeled RPE cells (*top* row, *white*) that was accompanied by the rapid and progressive recruitment of CX3CR1-GFP+ cells to the subretinal space (SRS)(*middle* row, *green*). Untreated age-matched animals were used as controls. Analysis of retinal sections at the same time-points (*bottom* row) revealed the appearance of CX3CR1-positive cells (*green*) in the SRS (*arrowhead*) beginning 1 day post-treatment and increasing in number thereafter. (**B**) Quantification of CX3CR1+ cells in the SRS showed a prominent increase in density following NaIO_3_ administration that peaked at 7 days post-treatment. (**C**) Immunohistochemical analysis of retinal flat-mounts from the same mice showed that in inner retinal lamina, NaIO_3_-induced RPE injury induced a biphasic change in microglial numbers in the inner retina. While the numbers of CX3CR1+ cells in the ganglion cell layer (GCL) demonstrated marked increases following 3 days post-injury, the numbers in the inner (IPL) and outer plexiform layers (OPL) demonstrated biphasic changes, initially decreasing down to near zero levels at 3 days, and then subsequently increasing at 7 and 30 days post-injury. (**D–F**) Quantification of the density of CX3CR1-positive cells in the GCL (**D**), IPL (**E**), and OPL (**F**) following NaIO_3_ administration. Scale bars = 60 µm. Column heights and error bars in **B,D,E,F** show mean ± SEM, ***indicates p < 0.0001; 1-way ANOVA, with comparisons made relative to uninjured control, n = 12 imaging fields from 3 male animals in each group.

To examine how RPE injury influences the distribution of myeloid cells in the retina, we employed the well-established model of NaIO_3_-induced injury in which systemic NaIO_3_ administration induces acute cellular damage in the RPE cell layer (Fig. 1A). The use of CX3CR1^GFP/+^ transgenic mice enabled the monitoring of changes in the distributions of CX3CR1-expressing myeloid cells in separate retinal lamina at different post-injury time-points (1, 3, 7, and 30 days) in flat-mounted and cryosectioned specimens. In the absence of injury, the outer retina of young adult animals is typically devoid of CX3CR1+ myeloid cells. However, following NaIO_3_-induced injury, CX3CR1+ cells emerged rapidly in the subretinal space (SRS) at 1d post-injury and increased significantly in density (to >500 cells/mm^2^) by 3d, remaining at that level for up to 30d (Fig. 1A,B). We also monitored time-dependent changes in myeloid cell density in the other retinal lamina; in the ganglion cell layer (GCL), CX3CR1+ cells increased significantly in density only from 3d post-injury and maintained that level for up to 30 days (Fig. 1C,D). In the inner and outer plexiform layers (IPL, OPL), CX3CR1+ cell density demonstrated an interesting biphasic change, decreasing first to very low levels 3d post-injury, and then subsequently increasing beyond baseline levels at 7–30d (Fig. 1C,E,F). These observations showed that injury centered on the RPE layer could induce prominent and time-dependent changes in the distribution of myeloid cells across the retina. These separate patterns of change in different retinal lamina suggested concurrent but separate modes of myeloid cell mobilization and/or recruitment in each locus.

### NaIO_3_-induced RPE injury induces initial mobilization of endogenous retinal microglia followed by infiltration of exogenous monocytes

**Figure 2:**
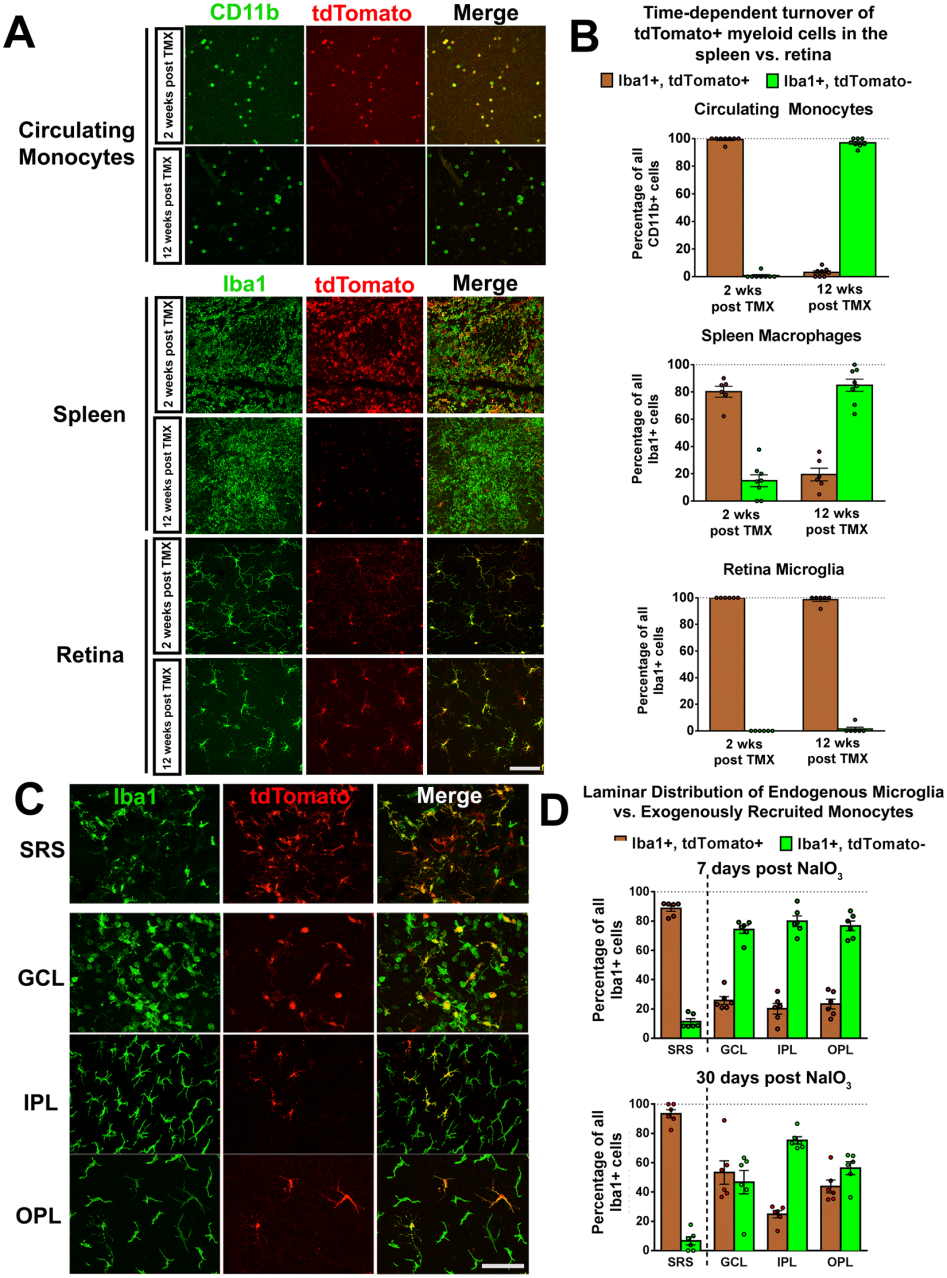
NaIO3-induced RPE injury induced early mobilization of endogenous retinal microglia from the inner retina to the subretinal space and the subsequent repopulation of the inner retina by infiltrating monocytes. (**A**) Use of the Cre-tdT transgenic mouse model enabled specific labeling of endogenous microglia in the retina, but not monocytes in the systemic circulation (i.e. “fate mapping” of microglia vs. monocytes). Young 2-month old CX3CR1^CreER/+^:Rosa26-flox-STOP-flox-tdTomato (Cre-tdT) transgenic mice were administered tamoxifen (TMX) by oral gavage (two doses 1 day apart, 500 mg/kg per dose) to induce the expression of tdTomato (*red*) specifically in CX3CR1-expressing myeloid cells, which were also marked by Iba1 or CD11b immunolabeling (*green*). At 2 weeks following tamoxifen induction, CD11b+ circulating monocytes (*top* row) isolated from the blood using flow cytometry cell sorting, uniformly co-expressed tdTomato. At 12 weeks following induction, following the time-dependent turnover of circulating monocytes, almost all CD11b+ cells were tdTomato-negative. The majority of Iba1+ myeloid cells in the spleen (*middle*) underwent a similar turnover between 2 and 12 weeks post-tamoxifen induction. By contrast, all Iba1+ microglia in the retina were tdTomato+ at both 2 weeks and 12 weeks after tamoxifen induction. (**B**) Quantification of the proportion of tdTomato+ among myeloid cells at 2 and 12 weeks in the blood, spleen, and retina. (**C**) At 12 weeks following tamoxifen induction, Cre-tdT transgenic mice were subjected to NaIO3-induced RPE injury and their retinas analyzed at 7 days post-NaIO_3_ in RPE and retinal flat-mounts. The predominant majority (90–100%) of Iba1+ myeloid cells in the subretinal space (SRS) were tdTomato+, indicating that they arise from endogenous microglia that have migrated from the inner retina. In contrast, only a minority (20–30%) of inner retinal Iba1+ microglia in the GCL, IPL, and OPL, were tdTomato+ at 7 days post-NaIO_3_, indicating that this population originates only partially from endogenous microglial sources, and consisted in large part of exogenously recruited, Iba1+, tdTomato- myeloid cells and their progeny. (**D**) Quantitation of endogenously derived retinal microglia (Iba1+, tdTomato+) and infiltrating myeloid cells (Iba1+, tdTomato-) in separate retinal laminae (SRS, GCL, IPL, OPL) at 7 days- (*top*) and 30 days- (*bottom*) post NaIO_3_ administration. Relative to cell counts at 7 days, the proportion of tdTomato+ cells in the SRS remained close to 100% at 30 days. However, exogenous tdTomato- cells constituted the majority population in the IPL and OPL at both 7 and 30 days. Column heights and error bars show mean ± SEM, n = 6 imaging fields from 3 male animals in each group. Scale bars = 60 µm. Column heights and error bars in B, and D show mean ± SEM, n = 6–8 imaging fields from 3 animals in each group.

As the sole use of CX3CR1-GFP expression does not distinguish whether changes in myeloid cell distribution resulted from either: (1) the migration and relocation of endogenous microglia within the retina, or (2) the recruitment of exogenous monocytes from the systemic circulation, we employed a method of “cell-fate mapping” that relied on the different rates of turnover in the two cell populations^29^. In these experiments, we administered a bolus dose of tamoxifen to two month-old CX3CR1^CreER/+^:Rosa26-flox-STOP-flox-tdTomato (Cre-tdT) transgenic mice to induce the activity of Cre recombinase, which resulted in the rapid expression of the fluorescent marker tdTomato in all CX3CR1-expressing myeloid cells. At two weeks following tamoxifen induction, the predominant majorities of circulating monocytes in the blood, macrophages in the spleen, and microglia in the retina all demonstrated expression of tdTomato (Fig. 2A,B). As monocytes have a limited tenure and are replaced with time by newly-derived cells, examination of the animals at 12 weeks post-tamoxifen administration demonstrated that the proportion of tdTomato+ monocytes approached 0%. This contrasted with that in long-lived retinal microglia which remained uniformly tdTomato+ at 12 weeks. This method of “cell fate-mapping” enabled the differential labeling of circulating monocytes (tdTomato-) vs. endogenous retinal microglia (tdTomato+).

Following this 12-week period, we subjected these “mapped” animals to NaIO_3_-induced injury and monitored the changing distributions of Iba1+, tdTomato- monocyte-derived cells from the systemic circulation and Iba1+, tdTomato+ endogenous microglia-derived cells in each retinal layer over time. We observed that at 7d post-injury almost all the myeloid cells in the SRS were tdTomato+, indicating that they consisted of endogenous microglia that have migrated from the inner retina (Fig. 2C,D). By comparison, the major proportions of myeloid cells in the GCL, IPL and OPL at 7 days following the recovery of cell numbers were tdTomato-, indicating that they had arisen from exogenous monocytes infiltrating the retina from the systemic circulation. These monocyte-derived cells appeared to take up niches previously occupied by endogenous microglia which were vacated when microglia migrated to the SRS soon after injury. At 30 days post-injury, the vast majority of myeloid cells in the SRS remained tdTomato+, indicating that migrated microglia form a stable population in this location, while in the inner retina, the myeloid cells continued to comprise of cells from both monocyte (tdTomato-) and microglial (tdTomato+) origins (Fig. 2D). At the 30d time-point, both monocyte- and microglia-derived myeloid cells in the IPL and OPL demonstrated ramified morphologies that were quantitatively similar between cells of either origin (Supplementary Fig. 1). Both groups of myeloid cells in the inner retina demonstrated immunopositivity for MHCII alloantigen I-A/I-E, CD45, and F4/80; differential levels of immunopositivity were not discernable between tdTomato- and tdTomato+ cells (Supplementary Fig. 2).

These data indicated that NaIO_3_-induced RPE cell injury attracted a large population of myeloid cells to the “immune-privileged” SRS whose origin arose predominantly from endogenous inner retinal microglia, with little contribution from exogenous monocytes. This prominent and rapid recruitment likely resulted in the short-term decrease in microglia numbers in the OPL and IPL observed at 1–3d post injury (Fig. 1C,E,F). This deficiency of microglia in the inner retina was then followed by a wave of infiltrating exogenous monocytes into the GCL, IPL, and OPL that likely entered the retina via the inner retinal vasculature, leading to the reconstitution of myeloid cell numbers in the IPL and OPL by 7d post-injury. These patterns demonstrate an initial dynamic mobilization of inner retinal endogenous microglia in response to RPE injury and a subsequent homeostatic response that repopulates the inner retina from both endogenous and exogenous sources.

### Recruitment of CCR2-expressing monocytes to the retina following NaIO_3_-induced RPE injury is restricted to the inner retina

**Figure 3:**
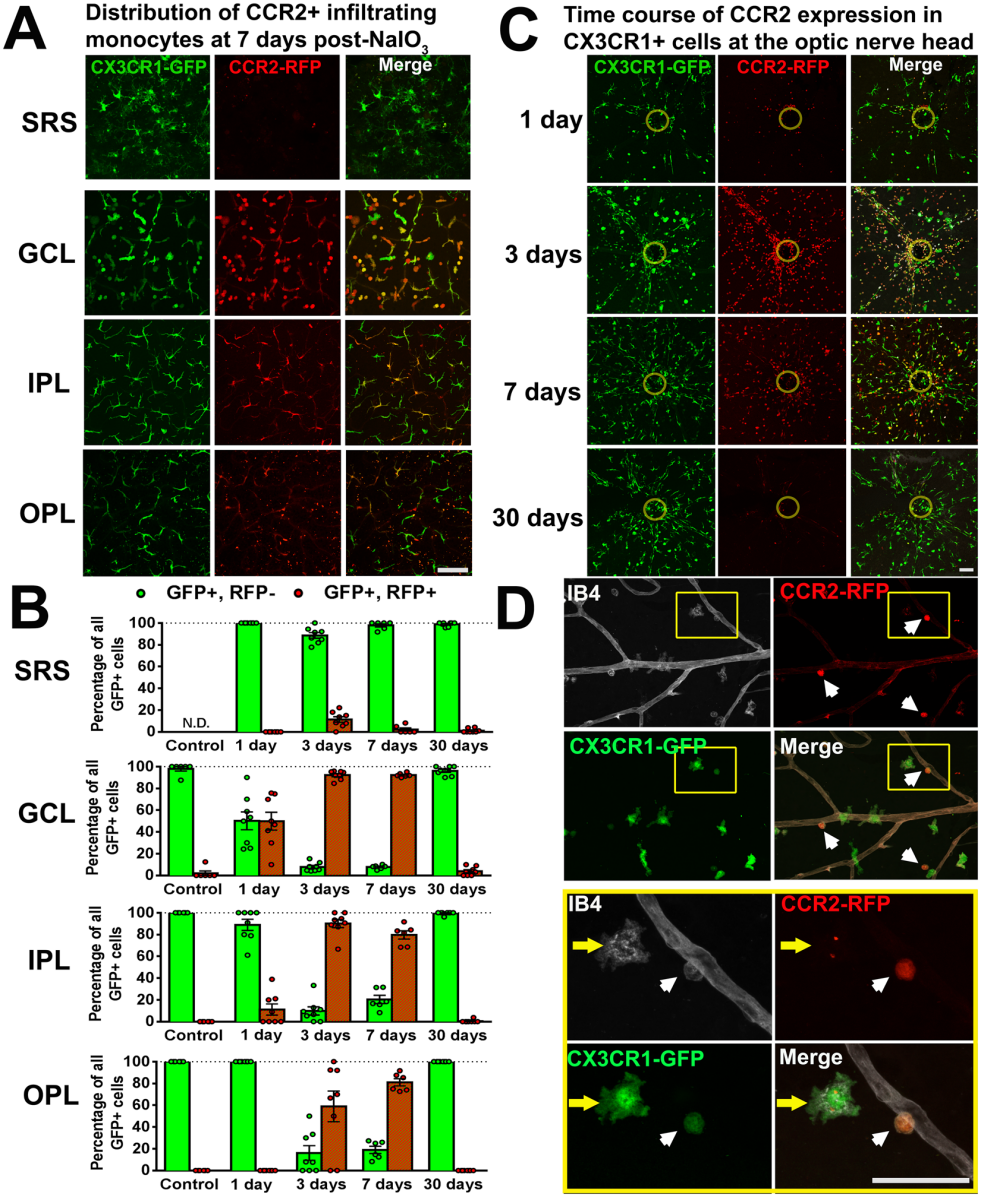
CCR2-expressing circulating monocytes infiltrate into the inner retina following NaIO3-induced RPE injury and express CCR2 transiently following infiltration. Adult CX3CR1^GFP/+^:CCR2^RFP/+^ transgenic mice were subjected to NaIO_3_-mediated RPE injury and analyzed at 0 (control), 1, 3, 7, and 30 days. RFP expression in CX3CR1+ cells enabled tracking of infiltrating monocytes in retinal flat-mounts. (**A**) At 7 days when the repopulation of the inner retina is underway, few or none of the CX3CR1+ cells in the subretinal space (SRS) were RFP+, indicating that infiltrating monocytes did not contribute to the SRS population. However, large proportions of CX3CR1+ cells in the GCL, IPL, OPL were RFP+, indicating that the recovery of myeloid cells in the inner retina derived from monocytic infiltration. The intensity of RFP fluorescence in CX3CR1+ cells appeared strongest in the GCL, and diminished in the IPL and OPL, suggesting that infiltrating monocytes downregulated RFP expression as they migrated into the deeper layers of the retina. (**B**) Quantitation of the proportion of CCR2-RFP+ cells among all CX3CR1-GFP+ cells in different retinal layers showed that CCR2-RFP+ cells appeared in the GCL at 1d, and in the IPL and OPL at 3d. CCR2-RFP expression in all retinal layers was transient, with minimal CCR2 expression in all retinal layers by 30 days. Column heights and error bars in B, and D show mean ± SEM, n = 8–12 imaging fields from 3 male animals in each group. (**C**) The intensity of RFP fluorescence and the concentration of RFP+ cells were greatest in the region of the optic nerve head (indicated by *yellow circle*), indicating it as the main entry point for monocytes. The time course of CCR2+ monocyte infiltration at this site demonstrated a peak in CCR2+ cell numbers at 3d, and decreased to very low levels by 30d, indicating that monocyte entry into the retina was transient, reaching its highest point when microglial densities in the IPL/OPL were at their nadir, and diminishing when IPL/OPL microglial densities recovered to their normal levels. (**D**) Morphology and vascular association of infiltrating CCR2 + monocytes. At 3 days, CCR2-RFP+ monocytes appeared as rounded cells (*white arrowheads*) in close proximity to retinal vessels (labeled with IB4, *white*), in contrast to nearby CX3CR1-GFP+, CCR2- cells (*green*) that were more ramified and less closely associated with the vasculature, suggesting that CCR2+ monocytes entered the retina by extravasation through the inner blood-retinal barrier as rounded cells, and then differentiated into microglia-like cells in the retina. Inset (*yellow box*) shows at high magnification a round extravasating CCR2+ monocyte (*white arrowhead*) partially within the lumen of retinal vessels. An adjacent CX3CR1+ cell located at a greater distance from the vasculature (yellow arrow) demonstrates decreased CCR2-RFP+ intracellular fluorescence and a more ramified morphology. Scale bars = 60 µm.

To corroborate the observations made using “cell fate-mapping”, we employed CX3CR1^GFP/+^:CCR2^RFP/+^ transgenic mice, in which CX3CR1-expressing and CCR2-expressing cells are identified by GFP- and RFP-fluorescence respectively. CCR2 is expressed by classical circulating monocytes, but not by endogenous microglia^30^, while CX3CR1 is expressed by both populations. We performed NaIO_3_-induced injury on 2-month old CX3CR1^GFP/+^:CCR2^RFP/+^ transgenic mice and followed the distribution of labeled myeloid cells at 1d, 3d, 7d, and 30d post-injury. We noted that across the entire post-injury time period (from 1 to 30 days post-injury), few to none of the CX3CR1-expressing cells in the SRS were CCR2-expressing (Fig. 3A,B), corroborating observations that the myeloid cells recruited into the SRS arose predominantly from migrating endogenous microglia which do not express CCR2. Prominent emergence of CCR2-expressing cells was observed in the GCL at 1d post-injury, and subsequently in the IPL and OPL at 3d post-injury, consistent with their initial entry at the GCL and their progressive infiltration into the deeper layers of the retina. However, at 30 days post-injury, almost none of the CX3 CR1-expressing myeloid cells in all retinal layers were CCR2-expressing, indicating that (1) the expression of CCR2 by infiltrating monocytes was transient and decreased following colonization of the retina, and that (2) the infiltration of exogenous monocytes into the inner retinal lamina was not sustained over time, and diminished as the inner retinal lamina became increasingly repopulated by myeloid cells.

We observed in retinal flat-mounts that infiltration of CCR2-expressing monocytes in the inner retina followed a center-to-peripheral gradient, showing the highest density near the large blood vessels at the optic nerve head (Fig. 3C); the numbers peaked at around 3d and gradually declined thereafter, with few new infiltrating cells at 30d. The entry of exogenous monocytes into the retina appeared to occur via extravasation through walls of retinal vessels in the form of rounded cells strongly positive for RFP (Fig. 3D). Myeloid cells located farther from retinal vessels tended to demonstrate a more ramified morphology and a lower expression of CCR2-RFP, suggesting that infiltrating monocytes undergo progressive “maturation” following entry into the retinal environment, gaining phenotypes observed in microglia cells such as ramified morphologies and decreased CCR2 expression.

### Recovery of myeloid cell numbers in the inner retinal lamina involves concurrent monocyte infiltration and proliferation of both monocytes and endogenous microglia

**Figure 4:**
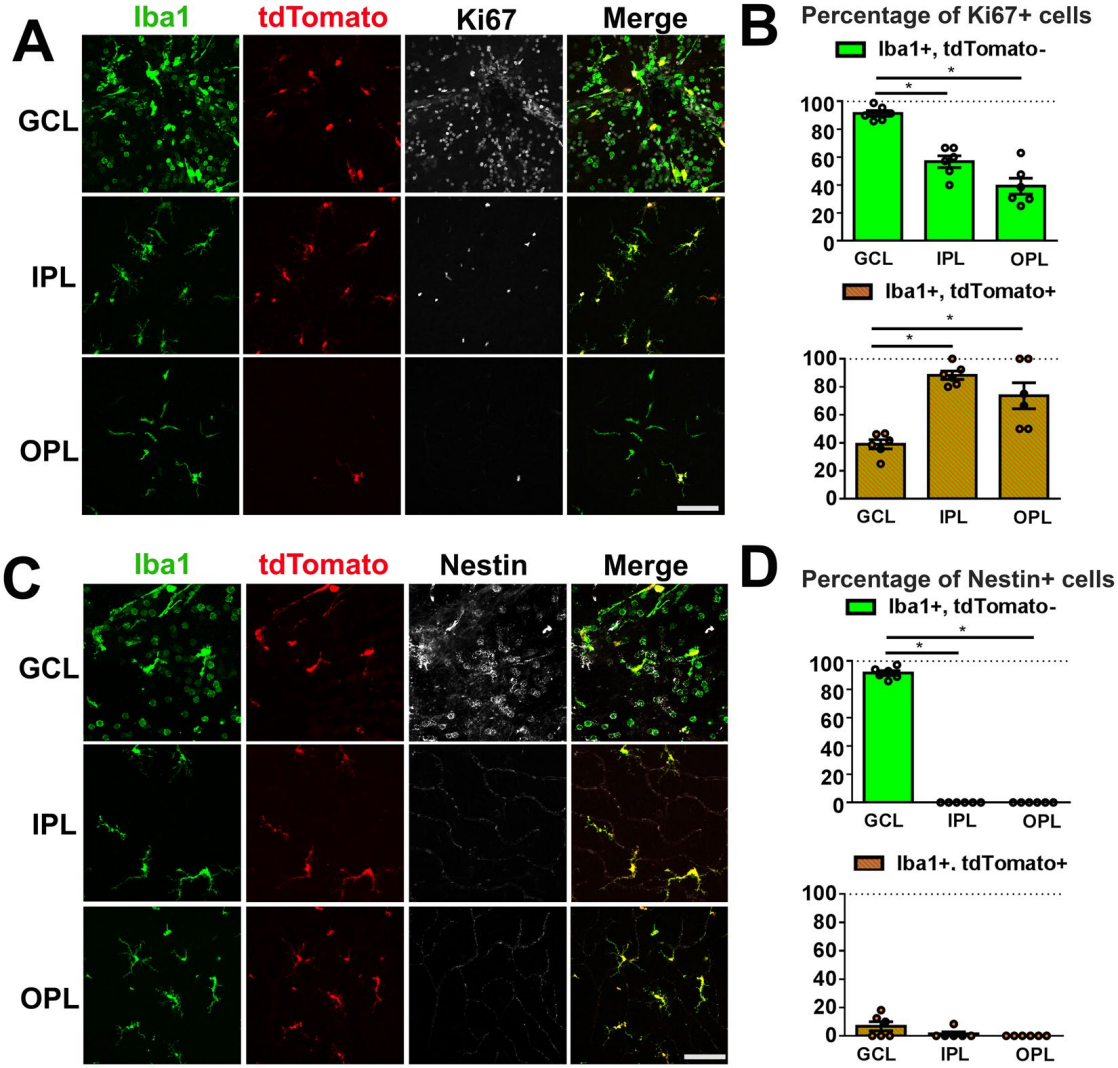
Repopulation of myeloid cells in the inner retina involves cellular proliferation by both endogenous microglia and infiltrating monocytes. (**A,B**) Cre-tdT transgenic mice in which endogenous microglia were previously labeled by tamoxifen-induced tdTomato expression were analyzed 2 days following NaIO_3_ injury with immunohistochemistry in retinal flat-mounts to Iba1 and Ki67 to detect proliferating myeloid cells. Both endogenous microglia (tdTomato+) and exogenously recruited monocytes (tdTomato-) demonstrated Ki67-immunopositivity in separate levels in the inner retina (GCL, IPL, OPL). While exogenously recruited monocytes (Iba1+, tdTomato-) had a high prevalence of Ki67-immunoposivity in the GCL, the prevalences were significantly lower among the monocyte-derived ﻿population﻿s in the IPL and OPL. Conversely, the prevalences of Ki67-immunopositivity among endogenous microglia (Iba1+, tdTomato+) were lower in the GCL, and comparatively higher in the IPL and OPL. (**C,D**) Nestin immunohistochemistry performed in parallel demonstrated that infiltrating monocytes (tdTomato-) were nestin+ upon entry in the GCL, but became nestin- as they migrated into the deeper retinal layers. Little or no nestin immunopositivity was observed in endogenous microglia (tdTomato+). Column heights and error bars in B and D show mean ± SEM, n = 2 imaging fields from each of 3 animals in each group. *Indicates p < 0.003; 1-way ANOVA. Scale bars = 60 µm.

Following the relocation of endogenous microglia from the inner retina to the SRS, the restoration of myeloid cell numbers in the IPL and OPL appears driven in part by monocyte entry to the retina from the circulation. To assess whether cell proliferation additionally contributes to this restoration, we performed immunohistochemistry for Ki67, a marker of cell proliferation, in “cell fate mapped” Cre-tdT mice to detect proliferating cells in both microglia and monocyte populations in the inner retina following NaIO_3_ injury. At 2d post injury, we found among infiltrating monocytes (Iba1+, tdTomato-) a very high (≈90%) prevalence of Ki67 immunopositivity in the GCL, and a lower (≈40–60%) prevalence in the IPL and OPL (Fig. 4A,B). Among endogenous retinal microglia (Iba1+, tdTomato+), we found that multiple cells were also Ki67+, with a lower prevalence in the GCL (≈40%) than in the IPL and OPL (≈70–90%). At this early post-injury time-point, when total numbers of myeloid cells in the inner retina were broadly decreased by mobilization to the SRS, homeostatic mechanisms appear to be triggered in the inner retina that activate concurrent (1) monocyte entry into the retina and (2) cellular proliferation in both monocyte- and endogenous microglia- populations, processes that are largely absent in the healthy adult CNS^10, 31^. The rate of proliferation in infiltrated monocytes appeared highest soon after entry in the GCL, while that in endogenous microglia was highest in IPL and OPL. In addition, immunohistochemistry to nestin, a progenitor marker previously described to be transiently expressed in infiltrating and proliferating myeloid cells^32^, was found to colocalize specifically to infiltrating monocytes (Iba1+, tdTomato−), but not to endogenous microglia (Iba1+, tdTomato+), post-injury (Fig. 4C,D). As nestin immunopositivity was found only in monocytes in the GCL, and absent in the IPL and OPL, it is likely that its expression was very transient, and was rapidly downregulated as the monocyte-derived cells migrated into the deeper retinal layers.

The contribution of monocyte infiltration and proliferation to myeloid cell repopulation of the inner retina was further corroborated in observations in NaIO_3_-injured CX3CR1^GFP/+^:CCR2^RFP/+^ transgenic mice that had been injected with BrdU consecutively for 3 days prior to mark proliferating cells. In this alternative method to detect proliferating cells, CCR2-expressing monocytes in the GCL demonstrated immunopositivity for both nestin and BrdU (Supplementary Fig. 3), confirming the involvement of actively proliferating exogenous monocytes in the overall injury response.

### Monocyte infiltration into the retina following RPE injury is regulated by CCR2 signaling

**Figure 5:**
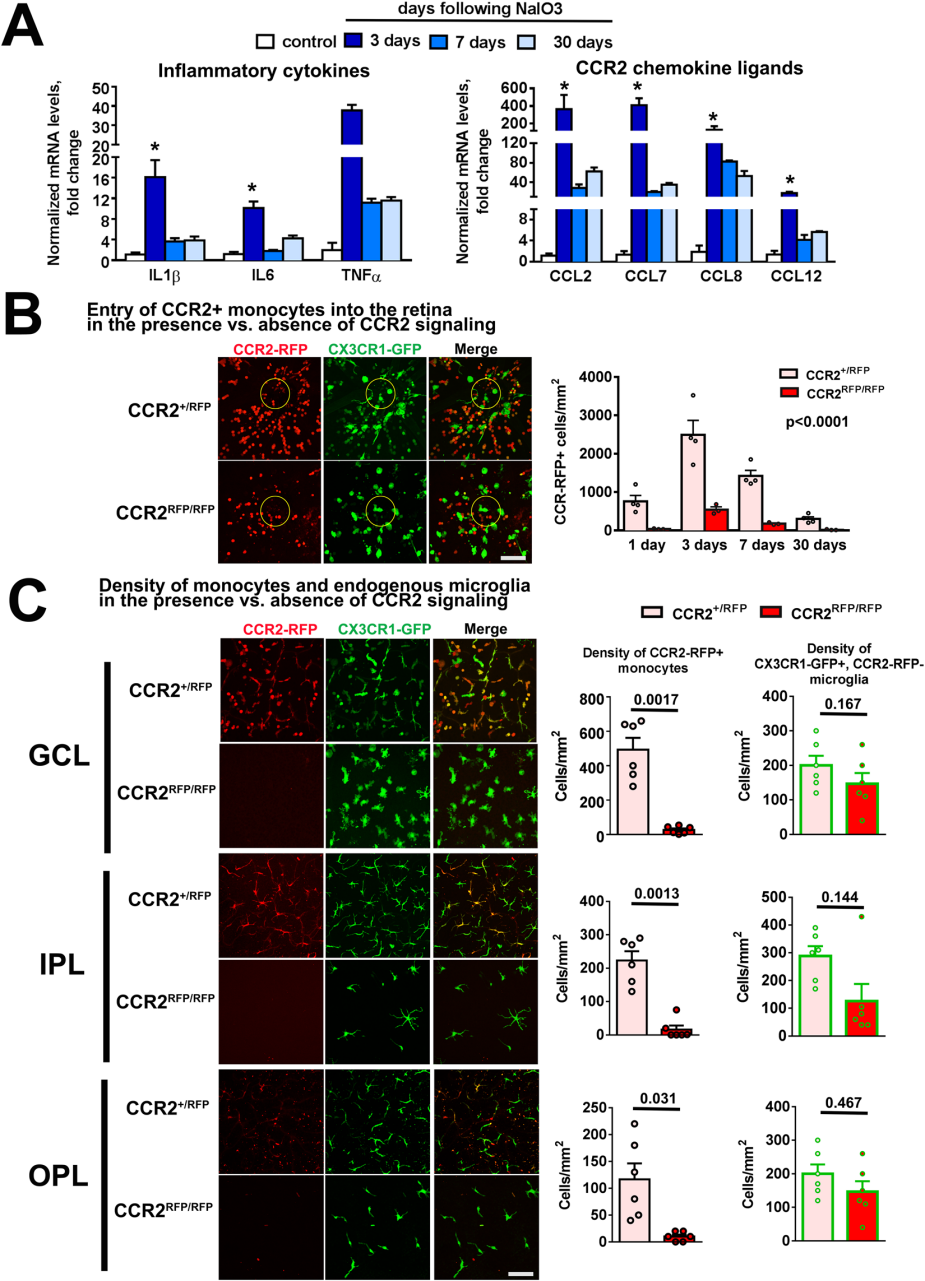
CCR2 signaling regulates monocyte entry and repopulation of the inner retina induced by RPE injury. (**A**) mRNA expression of inflammatory cytokines (IL-1β, IL6, and TNF-α) and chemokine ligands to CCR2 (CCL2, CCL7, CCL8, and CCL11) as measured by rtPCR were significantly upregulated 3 days following NaIO_3_-mediated RPE injury relative to uninjured controls and declined thereafter. The increases in expression were greatest for CCL2 and CCL7. (*Indicates p < 0.05 for comparisons relative to control, Kruskal-Wallis test with Dunn’s multiple comparisons test, n = 3 independent replicates per group.) (**B**) The entry of CCR2 + monocytes into the retina at the location of the optic nerve head (indicated by *yellow circle*) was monitored in CX3CR1^GFP/+^, CCR2^RFP/+^ and CX3CR1^GFP/+^, CCR2^RFP/RFP^ transgenic mice in which CCR2-mediated signaling was preserved and ablated respectively. In CCR2^RFP/+^ mice, CCR2+ monocytes appeared at the optic nerve at 1 day after NaIO_3_-mediated RPE injury, and peaked at 3 days before declining thereafter. In CCR2^RFP/RFP^ mice, changes in CCR2+ monocyte density followed the same temporal pattern but were significantly lower than those in CCR2^RFP/+^ mice (p < 0.0001, 2-way ANOVA, n = 3–4 animals for each time point). (**C**) The densities of monocytes in different retinal lamina were examined at 7 days in the mid-peripheral retina after NaIO_3_ administration. The numbers of exogenous CCR2+ monocytes in the GCL, IPL, and OPL were all significantly reduced in CCR2^RFP/RFP^ mice compared with CCR2^RFP/+^ mice (all p < 0.05; 1-way ANOVA, n = 6 animals of each genotype). However, the densities of CCR2-, CX3CR1+ myeloid cells, many of which comprise of endogenous microglia, were slightly but not significantly changed. Column heights and error bars in show mean ± SEM. Scale bar = 60 µm.

To investigate the mechanisms underlying myeloid cell homeostasis following RPE injury, we performed mRNA analysis in the retinas of NaIO_3_-injured CX3CR1^GFP/+^:CCR2^RFP/+^ mice at various times following injury. We found that mRNA levels of inflammatory cytokines IL1β, IL6, and TNFα were transiently upregulated following injury, peaking at 3d post-injury (Fig. 5A); mRNA levels of ligands to the CCR2 receptor, CCL2, CCL7, CCL8 and CCL12, were also significantly increased at 3d post-injury (Fig. 5B). Protein analysis of CCL2 and IL6 in retinal lysates were significantly increased following injury (Supplementary Fig. 4). Immunohistochemical analysis demonstrated that CCL2 and CCL7, the two most highly upregulated CCR2 ligands, were found in infiltrating monocytes, as well as neurons and/or macroglia in the GCL and INL (Supplementary Fig. 5). Given that the peak in CCR2-expressing monocyte infiltration was temporally coincident with CCR2 ligand upregulation at 3d post-injury, we investigated the role of CCR2 signalling in monocyte entry in NaIO_3_-injury by comparing injury responses in CCR2-sufficient CX3CR1^GFP/+^:CCR2^RFP/+^ mice vs. CCR2-deficient CX3CR1^GFP/+^:CCR2^RFP/RFP^ mice. While temporal changes in the numbers of RFP+ infiltrating monocytes at the optic nerve head were similar in pattern (peaking at 3d post injury and decreasing thereafter), the absolute numbers were significantly lower in CCR2^RFP/RFP^ compared with CCR2^RFP/+^ mice across all time points (p < 0.0001) (Fig. 5B). The mean densities of RFP+ monocytes at 7 days post-injury in the GCL, IPL, and OPL were also significantly lower in CCR2^RFP/RFP^ mice (Fig. 5C). However, the mean densities of endogenous microglia (GFP+, RFP−) were not statistically distinct between the genotypes. These findings indicate that while CCR2 signaling to monocytes induced by local retinal production of CCR2 ligands may positively regulate exogenous monocyte infiltration, the regulation of endogenous microglial numbers appeared independent of CCR2 signaling.

### Monocyte recruitment and microglial mobilization in an alternative genetic model of RPE injury follows a similar pattern as in NaIO_3_-mediated RPE injury

**Figure 6:**
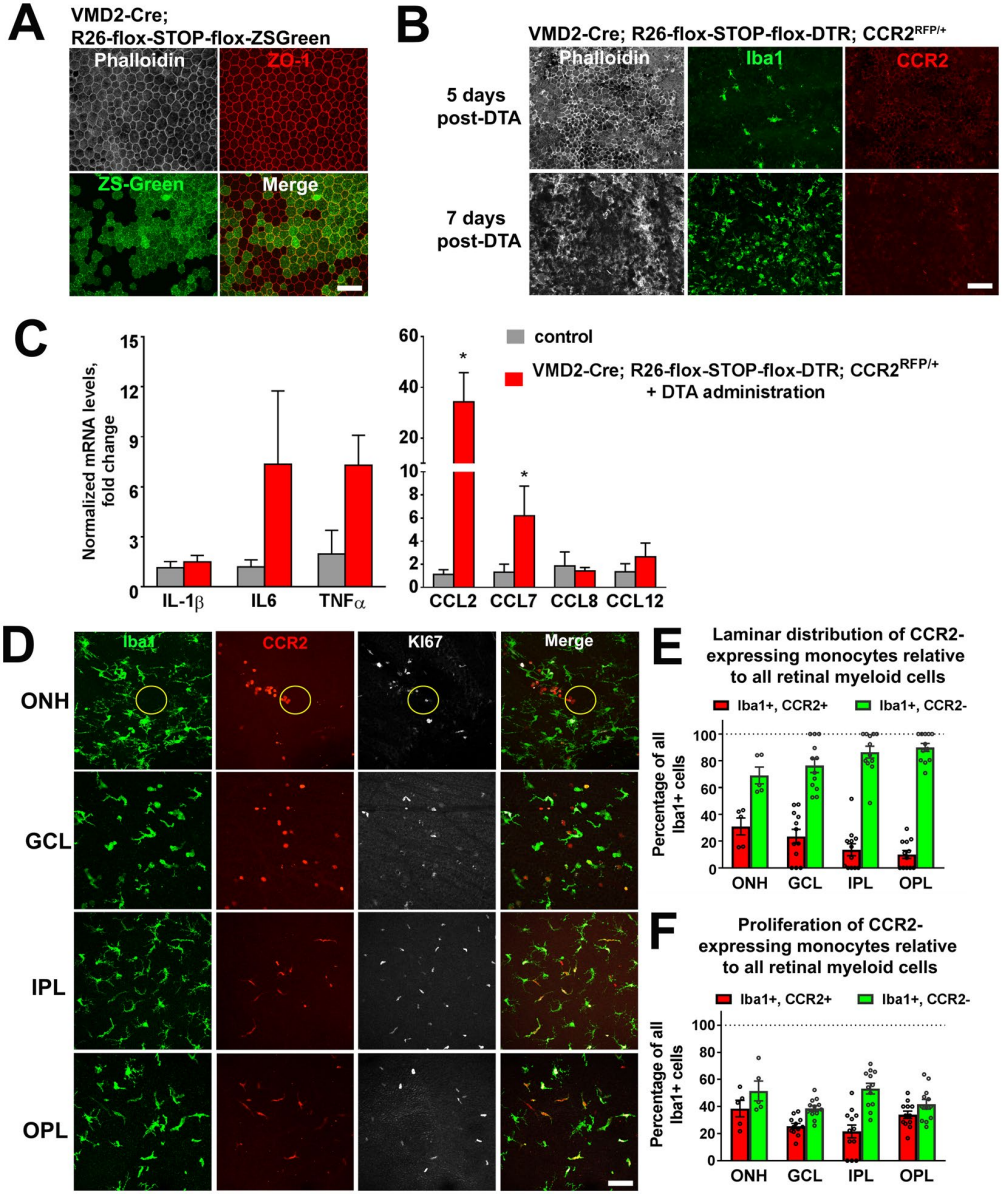
Microglia mobilization and monocyte recruitment in an alternative genetic model of RPE injury resemble responses to NaIO_3_-induced RPE injury. (**A**) Transgenic mice expressing Cre recombinase driven by the RPE-specific VMD2 promoter (VMD2-Cre) were crossed into the Rosa26-flox-STOP-flox-ZsGreen reporter strain. Resulting progeny demonstrated reporter expression in approximately 56% of RPE cells (identified by phalloidin (*white*) and ZO1 (*red*) staining in RPE flat-mounts). (**B**) VMD2-Cre mice in the CCR2-RFP background were crossed into the Rosa26-flox-STOP-flox-DTR strain to generate VMD2-Cre; Rosa26-flox-STOP-flox-DTR; CCR2^RFP/+^ mice which enabled (1) the RPE-specific expression of diphtheria toxin receptor (DTR) as well as (2) the tracking of CCR2-expressing monocytes. Intraperitoneal administration of diphtheria toxin A 2 days induced degeneration of a subset of RPE cells that was evident in phalloidin-stained RPE flat-mounts at 5 days post-DTA injection (*upper row*) and increasing in extent at 7 days post-DTA injection (*bottom row*). Iba1 staining (green) revealed a prominent recruitment of endogenous microglia to the subretinal space with little or no recruitment of CCR2-RFP+ monocytes. (**C**) mRNA expression of inflammatory cytokines, IL6, and TNF-α, and chemokines CCL2 and CCL7 as measured by rtPCR were upregulated 3 days following DTA administration in VMD2-Cre; Rosa26-flox-STOP-flox-DTR; CCR2^RFP/+^ mice relative to untreated control mice. Column heights and error bars show mean ± SEM; *indicates p < 0.05; Mann-Whitney test, n = 3–5 independent replicates per group. (**D**) Analysis of the inner retina of VMD2-Cre;R26-flox-STOP-flox-DTR; CCR2^RFP/+^ mice 7 days after DTA injection showed the infiltration of CCR2+ monocytes in areas near the optic nerve head (ONH) (*yellow circle*), as well as in the GCL, IPL, and OPL layers. In the inner retina, Ki67 immunopositivity was found in both infiltrating CCR+ monocytes as well as endogenous CCR2- microglia, demonstrating that genetic induction of RPE injury, as in the NaIO_3_ –induced model of RPE injury, involved the mobilization of endogenous microglia and the exogenous recruitment of systemic monocytes to the inner retinal lamina. (**E**) Quantitation of infiltrating monocytes (Iba1+, CCR2+) and endogenously derived retinal microglia (Iba1+, CCR2-) in separate retinal locations 7 days after DTA injection. (**F**) Quantitation of the proportion of proliferating Ki67+ infiltrating monocytes (Iba1+, CCR2+) and endogenously derived retinal microglia (Iba1+, CCR2-) in separate retinal loci, showing proliferative activity of myeloid cells from both sources. Scale bars = 60 µm.

To investigate whether mechanisms of monocyte recruitment and microglial mobilization were peculiar to NaIO_3_-induced injury or applicable to RPE injury more generally, we performed experiments in an alternative mouse model of RPE injury. In this model, transgenic VMD2-Cre; Rosa26-flox-STOP-flox-DTR mice, which expressed Cre recombinase driven by the RPE-specific VMD2 promoter (VMD2-Cre), enabled the expression of the receptor for diphtheria toxin (DTR) specifically in RPE cells. To confirm that Cre recombinase was expressed specifically in the RPE cells, VMD2-Cre mice were crossed with a reporter strain to generate VMD2-Cre: Rosa26-flox-STOP-flox-ZsGreen mice; we found in these mice that Cre recombinase activity was induced in about half (56%) of all RPE cells (Fig. 6A). To track monocyte entry into the retina, we crossed VMD2-Cre mice to CCR2^RFP/RFP^ mice to generate VMD2-Cre: Rosa26-flox-STOP-flox-DTR: CCR2^RFP/+^ mice. Two-month old transgenic mice were administered diphtheria toxin A (DTA) by intraperitoneal injection to induce RPE degeneration. Patchy disorganization of the RPE layer was observed at 5 days post-DTA, which increased to involve RPE degeneration at 7 days post- DTA (Fig. 6B). Comparatively, these RPE degenerative changes were slower and less severe than for NaIO_3_-induced injury. Similar to NaIO_3_-induced injury, we observed an emergence and progressive increase in Iba1+ myeloid cells in the subretinal space following DTA administration; these were uniformly negative for CCR2, indicating that they originated from endogenous retinal microglia, and not infiltrating monocytes. We also noted that mRNA expression levels for inflammatory cytokines (IL6, TNFα) and CCR2 ligands (CCL2, CCL7) were increased 3 days post-DTA administration (Fig. 6C), similar to those found in NaIO_3_-induced injury, albeit to lower extents. Histological examination at 7 days post-DTA administration revealed the presence of both CCR2-expressing monocytes and CCR2-negative microglia at each lamina of the retina (Fig. 6D,E), indicating the presence of infiltrating monocytes in the inner retina. Immunohistochemical analysis revealed Ki67 immunopositivity in CCR2-expressing monocytes and CCR2-negative microglia (Fig. 6D,F), indicating that proliferation in both cell populations and in all retinal loci contributed to overall myeloid cell homeostasis in the retina. These findings together indicate that the patterns of myeloid cell redistribution and recruitment were similar to those in NaIO_3_-induced injury, consisting of (1) early endogenous microglia mobilization to the SRS space towards RPE injury, (2) entry of exogenous monocytes into the inner retina, and (3) proliferation of monocytes and endogenous microglia in the inner retina.

### Monocyte recruitment and microglial mobilization with retinal aging

**Figure 7:**
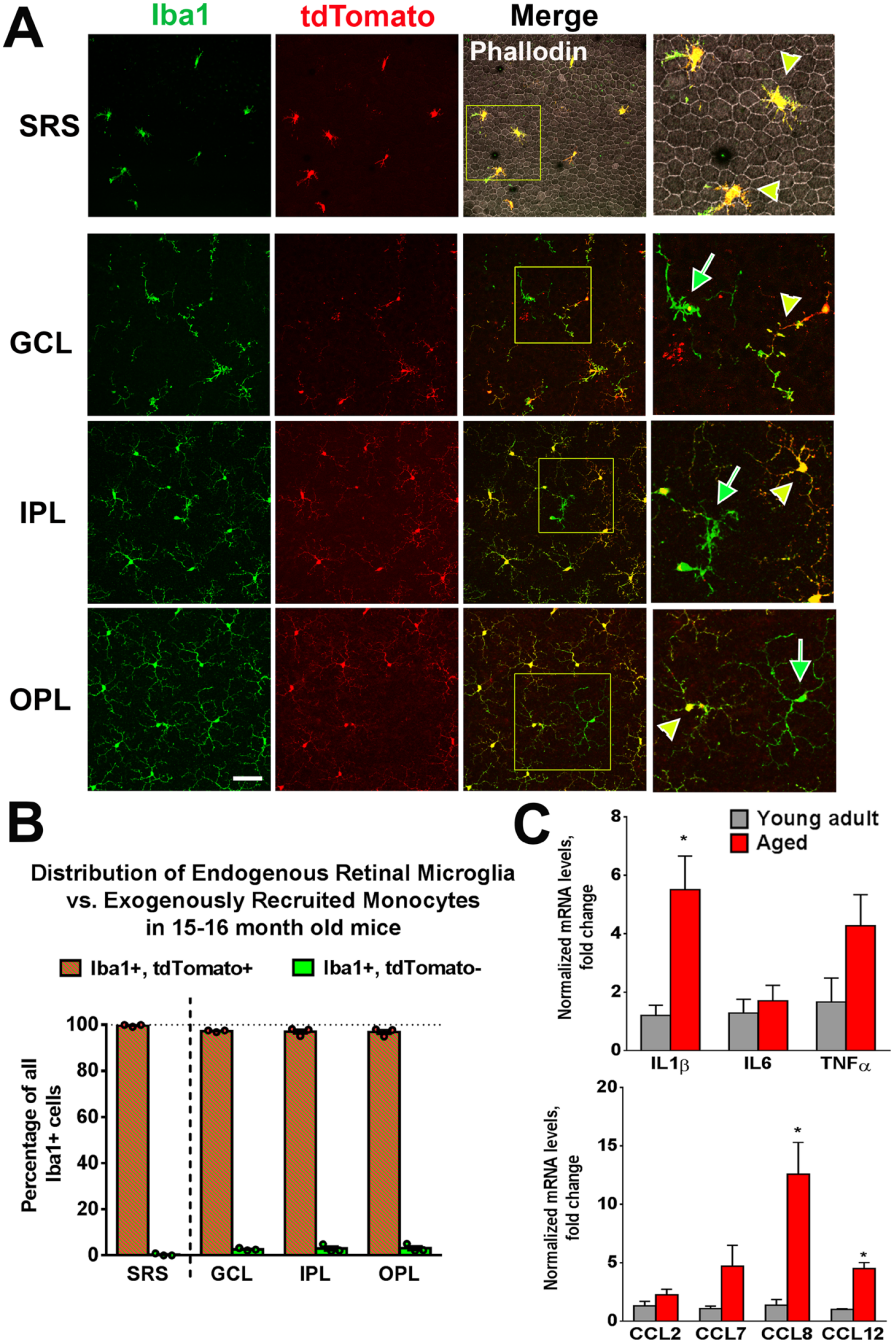
Microglia mobilization and monocyte recruitment during aging in the mouse retina. (**A**) Cre-tdT mice were administered tamoxifen at 2 months of age to enable “cell-fate” mapping of retinal microglia vs. systemic monocytes, and then allowed to undergo normal aging under standard conditions in the absence of injury until analysis at 15–16 months of age. (*Top row*) Analysis in sclerochoroidal-RPE flat-mounts demonstrated the presence of Iba1+ myeloid cells in the subretinal space (SRS) on the apical surface of RPE cells (labeled with phalloidin). All Iba1+ cells in the SRS were almost entirely tdTomato+ (*yellow arrowheads*), indicating that they had arisen from endogenous microglia. (*Lower rows*) Iba1+ cells in the inner layers (GCL, IPL, OPL) were predominantly tdTomato+ (*yellow arrowheads*); however a very small ( < 5%) minority of Iba1+ cells was tdTomato-negative (*green arrows*), indicating their origin from infiltrating monocytes. Scale bar = 100 µm. (**B**) Quantification of Iba1+, tdTomato+ cells (from endogenous microglia) and Iba1+, tdTomato- cells (from infiltrating monocytes) in all retinal lamina, demonstrating very low levels of contribution of exogenous monocytes to the resident population of retinal myeloid cells. (**C**) Comparison of mRNA levels of inflammatory cytokines and CCR2 ligands as measured by qRT-PCR between retinas from young adult (2 months old) and aged adult (24 months old) mice. Column heights and error bars show mean ± SEM; *indicates p < 0.05; Mann-Whitney test, n = 3–5 independent replicates per group.

In the absence of injury, CX3CR1- and Iba1-expressing myeloid cells have been documented to accumulate in the SRS with aging, increasing in density as a monotonic function of age^33, 34^. However, it is unclear whether these subretinal myeloid cells originated from the migration of intraretinal microglia or from exogenous infiltration of monocytes. To examine this, we performed “cell-fate mapping” of microglia vs. monocytes in Cre-tdT transgenic mice at 2 months of age, and then maintaining these animals in the absence of injury under standard housing conditions until 15–16 months of age. Histological analysis revealed that virtually all Iba1+ myeloid cells in the SRS were tdTomato+ (Fig. 7A), indicating that they had originated from endogenous retinal microglia that had migrated from the inner retina. In the inner retinal lamina (GCL, IPL, and OPL), while the majority of Iba1+ cells were tdTomato+, a small minority (<5%) of cells were Iba1+, tdTomato-, indicating that a low level of monocyte infiltration from the systemic circulation occurred under normal aging conditions (Fig. 7B). Isolated, rare, Ki67-labeling was also detected among Iba1+, tdTomato+ cells, indicating a very low level of replication in endogenous microglia during normal aging (data not shown). Comparison of the levels of mRNA expression of inflammatory cytokines and CCR2 ligands between young (2 month-old) and aged (24 month-old) animals also showed a general age-dependent increase in these factors (Fig. 7C), albeit to a considerably lower extent than in the two models of RPE injury. Taken together, these results indicate that mobilization of endogenous microglia from the inner retina to the subretinal space occurs with aging as with RPE injury but to a much lower extent. This is also accompanied by a low level of exogenous monocyte infiltration that may contribute to maintaining, or even increasing,the density of microglia in the inner retina with aging^35^.

This long-term stability of mobilized myeloid cells was also observed in infiltrating monocytes that had been recruited into the retina following sodium iodate-induced RPE injury. We observed that myeloid cells in the inner retina that had originated from infiltrating monocytes remained present six months following injury, maintaining a ramified morphology, and demonstrating little proliferative activity, as evidenced by low rates Ki67-positivity (Supplementary Fig. 6). These observations indicate that homeostatic mobilizations of myeloid cells occurring in aging and in injury responses can result in fairly-stable, long-lived cell populations in the regions colonized.

## Discussion

### Relationship between RPE injury and retinal myeloid cell redistribution in age-related macular degeneration and aging

The effects of RPE injury and aging on innate immune cells in the retina are of translational interest as they are relevant to pathobiology of age-related macular degeneration (AMD). AMD, the largest cause of vision loss in older adults in the developed world^36^, involve on one hand, RPE disorganization and degeneration as hallmark features^37^, and on the other, immune mechanisms as etiologic factors^38, 39^. The innate immune system has been implicated in particular as myeloid cells have been described to accumulate in the subretinal space near areas of RPE degeneration in both mouse models of AMD^19, 40^, and in human histopathological specimens^15–18^. In addition, while myeloid cells are nearly absent from the outer layers of the young healthy retina, they increase in number in the subretinal space with aging^33, 34^, augmenting their physical interactions with RPE cells. These findings indicate that myeloid cell-RPE interactions, particularly in the aged retina, are important in AMD pathogenesis^21^, and have prompted clinical investigations into immunomodulatory approaches for the treatment of AMD^41–43^. However, details on how RPE injury influences the number and distribution of myeloid cells in the retina, and the sources from which these myeloid cells arise, are still lacking, limiting the interpretation of interventional studies and the formulation of new immunomodulatory strategies.

In this study, we employed pharmacological and genetic methods to induce RPE injury in transgenic mouse models in which different myeloid cell populations are differentially labeled to examine resulting changes in microglia and monocyte distributions in a time-dependent manner. In particular, the NaIO_3_ injury model, which induces RPE necroptosis^44, 45^, has been used in multiple studies as a model for AMD-relevant RPE damage^46–48^. Our models aimed to simulate the advanced atrophic (geographic atrophy) form of AMD by inducing primary RPE damage that is associated with ensuing photoreceptor loss, however it should be noted that additional AMD-relevant phenotypes of drusen regression and gradual expansion of areas of atrophy are not recapitulated in these or other existing models^49^. Our findings demonstrate that myeloid cell responses to RPE injury are dynamic and occur in stages, consisting of (1) an early mobilization of endogenous microglia from the inner retina to the RPE layer, and (2) a subsequent homeostatic response to replenish microglia in the inner retina via monocytic infiltration and local proliferation. We also performed parallel studies in the aging retina showing that myeloid cell accumulation in the subretinal space with aging similarly originated from endogenous microglia, with very limited contribution from infiltrating monocytes.

### Mobilization of endogenous retinal microglia to the subretinal space with RPE injury

These findings here suggest that the accumulation of microglia in the subretinal space following RPE injury and during aging are induced by apically-directed signals arising from RPE cells that impinge on endogenous microglia within the retina. We were not able to detect evidence of significant infiltration of circulating monocytes via the choroidal vasculature into the outer retina, even though the RPE is not impermeable to monocyte infiltration, as monocyte infiltration into the subretinal space had been previously described in models of inherited photoreceptor degeneration^50^ and choroidal neovascularization^51^. As such, subretinal myeloid cells may arise variably from either intraretinal microglia or from exogenous monocyte sources depending of the specific context of each injury type, as determined by the nature of the outer retinal pathology and the chemoattractive signals released. Our observations here indicate that myeloid cells recruited to areas of RPE disruption in AMD-associated lesions can arise from endogenous retina microglia. As such, it may be postulated that interventions that inhibit RPE-microglia chemoattractive signaling within the retina in the form of agents delivered locally to the eye may be effective in reducing myeloid cell numbers in the outer retina, thereby decreasing deleterious microglia-RPE^20^ and microglia-photoreceptor interactions^52^ implicated in AMD-related degeneration. Future development of immunohistochemical markers that can potentially distinguish microglia vs. monocytes^53, 54^ will be helpful in verifying the origin of recruited myeloid cells to the RPE layer in histopathological studies of AMD.

We observed that the recruitment of endogenous microglia from the inner retina to the subretinal space occurs very rapidly upon NaIO_3_-induced RPE injury, and result in a persistent population of microglia in the subretinal space. As these subretinal Iba1+ cells do not show CCR2 expression at any point in time, they are unlikely to involve CCR2+ systemic monocytes that have been recruited into the outer retina via chemoattractive CCR2 ligands. The precise identities of RPE-derived signals responsible for the recruitment of endogenous microglia to the subretinal space are yet unelucidated; candidate mechanisms include chemokine signaling^55^ and/or ATP-mediated signaling to microglia via purinergic receptors^56, 57^, which may constitute potential targets for intervention.

### Homeostatic repopulation responses in the inner retina following RPE injury

Although the inner retina was not a direct site of cellular damage in our two RPE injury models employed here, we observed a prominent infiltration of systemic monocytes into the inner, but not the outer retina, soon after endogenous microglia were recruited from the inner retina into the subretinal space. Monocytic entry into the retina appeared to be centered most prominently near the optic nerve, with monocytes extravasating through walls of retinal vessels. Infiltrating monocytes at the point of entry demonstrated a rounded morphology, expressed nestin and CCR2, and were highly proliferative. As they moved farther into the retina away from blood vessels, they developed a more ramified morphology, downregulating nestin and CCR2 expression at the same time. Monocytic infiltration into the retina appeared limited in duration; 30 days after RPE injury, when microglial density in the inner retina had been restored, further entry of CCR2+ monocytes into the retina diminished. Infiltrated monocytes also acquired morphologies that were indistinguishable from neighboring endogenous microglia. These observations led us to interpret monocytic infiltration in this context as being not being directly driven by RPE injury signals, but instead induced as a homeostatic response to microglial depletion in the inner retina following the outward migration of endogenous microglia. This infiltration of monocytes appears to result in the restoration of a population of uniformly spaced and ramified myeloid cells in the inner retina, likely as an adaptive response to the requirement of microglia in the plexiform layers for healthy retina function. Our previous work has demonstrated that the long-term deficit of microglia in the inner retina results in synaptic degeneration and the loss of electrophysiological responses to light stimuli^3^, indicating the need for myeloid cell homeostasis and maintenance functions in the retina.

This homeostatic response to restore inner retinal myeloid cell numbers was not constituted solely by monocytic responses but also accompanied by an increased proliferative response in residual endogenous microglia in the inner retina. These observations indicate that under conditions of microglia depopulation, endogenous microglia can transition to a more proliferative phenotype, collaborating with infiltrating monocytes to restore intraretinal myeloid cell numbers. Although endogenous microglia and infiltrating monocytes can demonstrate different phenotypes in other injury models (e.g. light injury, radiation followed by bone-marrow transplantation) during the peri-injury period^29^, it is unclear in our model systems whether neighboring myeloid cells, derived separately from endogenous microglia and exogenous monocytes, may be phenotypically distinct or similar following the re-establishment of homeostasis. On histological analysis, we were not able to distinguish the two populations except by cell-fate mapping as they demonstrated similar morphologies, were integrated into the same regular horizontal mosaic arrangement, and were uniformly negative for nestin, CCR2, and proliferation markers. While a previous study using different injury models^29^ have reported that retinal myeloid cells derived from microglia and exogenous monocytes expressed differential levels of cell surface markers as analyzed by flow cytometry, these different levels of expression were not apparent on immunohistochemical analyses in our model of NaIO3 injury. The similarities between the two groups of cells seen in our model suggest that they may contribute to the constitutive functions of retinal microglia in similar ways, although this requires further study and verification.

The recruitment of CCR2+ monocytes into the retina as a homeostatic response to the relocation of endogenous microglia highlights a novel function for monocyte infiltration into the CNS. Previous studies of various CNS injury models have focused on monocyte recruitment directly to the site of injury; depending on the injury model, the infiltrated monocytes have been noted to contribute significantly to the neuroinflammatory response^58^ and to neurodegeneration^59, 60^, or on the contrary, help resolve inflammation^61^ and aid functional recovery from injury^62^. Infiltrated monocytes have been noted to have a limited tenure in the CNS in some models^63^ and establish themselves more permanently in others^64^. Interestingly, in our models of RPE injury, monocyte recruitment is directed to zones of microglial depletion in the inner retina, rather than zones of injury in the outer retina. As such, this recruitment of monocytes more likely represents a mechanism subserving myeloid cell homeostasis in the neural parenchyma, rather than being involved in the potentiation of inflammation. The recruited monocytes in our models also have an extended tenure in the inner retina; evidence of Iba1+, tdTomato- monocyte-derived cells can be still located in the retina 6 months following RPE injury.

The existence of an innate drive to maintain microglial homeostasis in the CNS has been recently demonstrated by studies in which depletion of the endogenous pool of CNS microglia using genetic and pharmacological methods induced a prominent repopulation response that restores resident myeloid cell populations^60, 65, 66^. In these studies, no concurrent tissue injury was inflicted and no compromise of the blood-brain barrier was induced; the source of repopulating cells was also thought to come from endogenous cells within the CNS, postulated to be either resident progenitor cells that differentiate into microglia^66^ or endogenous residual microglia that replicate to regenerate the full complement of cells^65^. In either case, contributions by infiltrating monocytes were found to be absent. In our models of RPE injury, we observed a combined contribution of both infiltrating monocytes and proliferating residual microglia occurring concurrently with an increase in the retinal expression of inflammatory cytokines and CCR2 ligands. The presence of these upregulated factors may facilitate monocyte entry and chemotaxis into the retina, enabling them to participate in the repopulation response. The diminished monocyte recruitment in CCR2-deficient mice also suggests a contribution of CCR2-mediated signaling in the homeostatic recruitment of exogenous monocytes, as has been found for the CCR2-regulated recruitment of monocytes to the peritoneum in a model of peritonitis^67^. However, the differences in monocyte recruitment in CCR2-deficient animals have to be considered together with a reduction of circulating monocytes in these animals^68, 69^, which can in itself cause to decreased monocyte recruitment to the retina. By comparison, in healthy aged mouse retinas, the expression  levels of inflammatory cytokine and CCR2 ligands were however only slightly elevated relative to that in young retina, and concordantly, inner retinal microglia homeostasis was driven primarily by endogenous microglial proliferation, with minimal exogenous monocyte recruitment. Together, these findings indicate that the source of “replacement” myeloid cells to take the place and function of endogenous microglia in the CNS in disease and aging can arise from a number of sources that include CCR2+ monocytes and/or endogenous microglia, depending in part on the levels and nature of intraretinal mediators produced and the preservation of the blood-retina barrier.

In summary, we find here that direct response and chemotaxis of myeloid cells to RPE injury are constituted primarily by endogenous microglia recruited locally from the inner retina. This mobilization of endogenous retinal microglia then induces a secondary adaptive response consisting of exogenous monocyte recruitment and microglial proliferation that repopulates the myeloid cell population in the inner retina. These patterns of coordinated microglial mobilization and monocyte infiltration in the mouse retina in response to RPE injury provide us a useful perspective towards the goal of targeting innate immune cells in AMD treatment and prevention. One translational extrapolation from the findings here may be that immunomodulation in AMD therapy may more suitably be targeted to the eye in a local manner towards the injury responses of endogenous microglia resident within the retina. Broad systemic immunosuppression of monocyte response may have the consequence of inhibiting systemic monocyte recruitment that constitutes an adaptive response to maintain microglial homeostasis. Clarifying the identity and physiology of participating myeloid cells in retinal injury will enable the design of more precise and targeted interventions that limit deleterious immune reactions while preserving adaptive endogenous functions of the innate immune system.

## Electronic supplementary material

Supplementary Data
