# Supplementary material for: Monocyte infiltration and proliferation reestablish myeloid cell homeostasis in the mouse retina following retinal pigment epithelial cell injury
Source: Sci Rep. 2017 Aug 16;7:8433. doi: 10.1038/s41598-017-08702-7 (PMC5559448; doi:10.1038/s41598-017-08702-7)
Supplement: Supplementary file 1 — Supplementary Data [file 41598_2017_8702_MOESM1_ESM.pdf]

Supporting Information for:

Monocyte infiltration and proliferation reestablish myeloid cell homeostasis in the mouse retina following retinal pigment epithelial cell injury

Wenxin Ma[1], Yikui Zhang[1], Chun Gao[2], Robert N. Fariss[2], Johnny Tam[3], Wai T. Wong[1*]

[1]Unit on Neuron-Glia Interactions in Retinal Disease, [2]Biological Imaging Core, National Eye Institute, [3]Ophthalmic Genetics and Visual Function Branch, National Eye Institute, National Institutes of Health, Bethesda, MD 20892, USA.

Filename: **Supporting Information for Ma et al.,**

Brief Description of Material:

- Supplementary Figures 1-6
- Supplementary Table 1

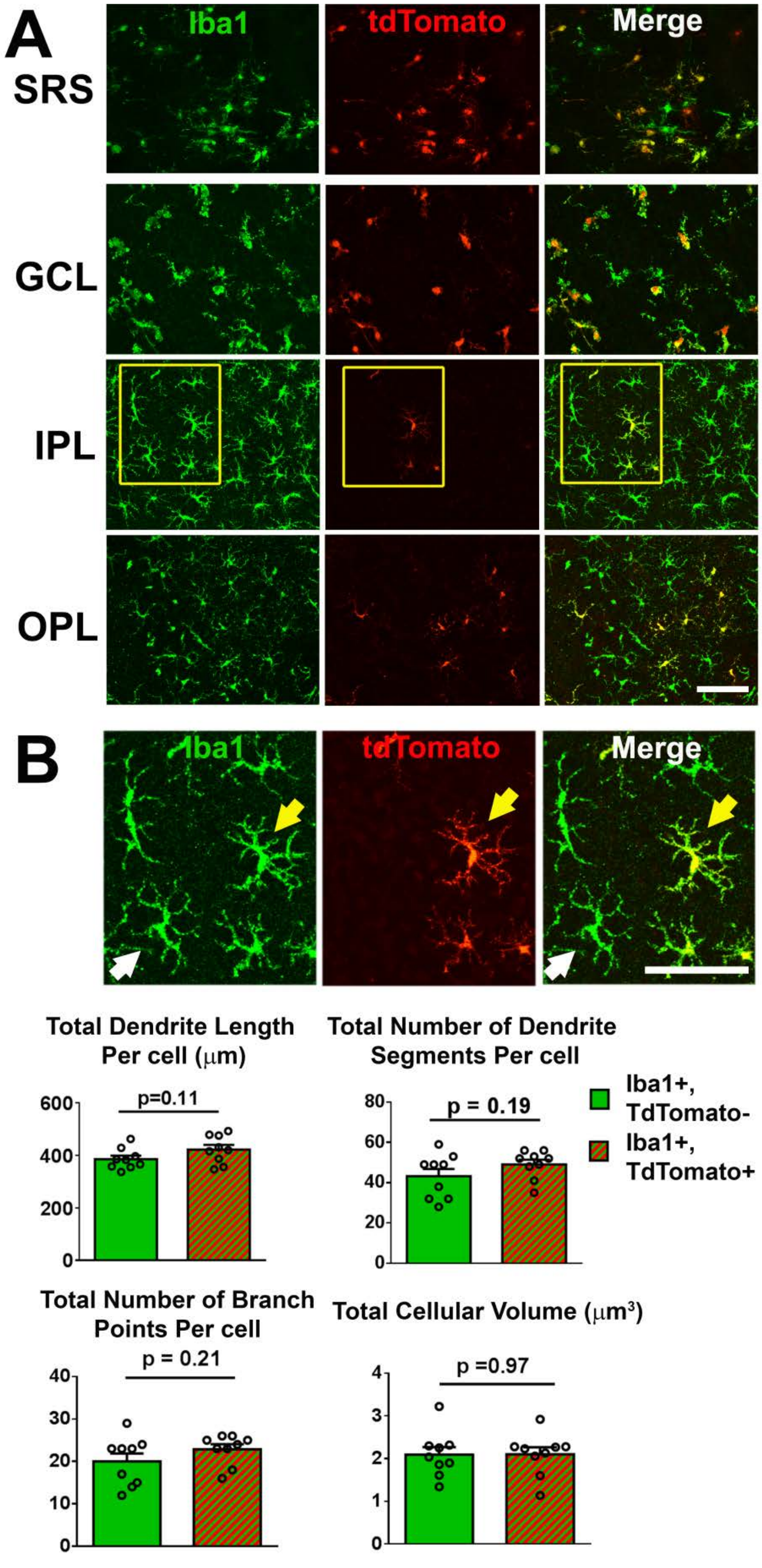

A
Iba1
tdTomato
Merge
SRS
GCL
IPL
OPL
B
Iba1
tdTomato
Merge
Total Dendrite Length Per cell (μm)
p=0.11
600
400
200
0
Total Number of Dendrite Segments Per cell
p = 0.19
80
60
40
20
0
Iba1+, TdTomato-
Iba1+, TdTomato+
Total Number of Branch Points Per cell
p = 0.21
40
30
20
10
0
Total Cellular Volume (μm3)
p =0.97
4
3
2
1
0

Supplementary Figure 1. Infiltrating monocytes recruited to new locations in the inner retina following $NaIO_3$-mediated RPE injury have extended tenures and develop microglia-like ramified morphologies over the course of 1 month. (**A**) Three months following tamoxifen-mediated Cre recombination to "fate-map" endogenous retinal microglia with tdTomato, Cre-tdT transgenic mice were subjected to $NaIO_3$-mediated RPE injury. Thirty days following RPE injury, retinal tissues were analyzed using RPE and retinal flat-mounts. Iba1+ cells in the subretinal space (SRS) remained predominantly tdTomato+, indicating that this population of endogenous microglia migrated from the inner retina was relatively persistent over a period of 1 month. The majority of cells in the IPL and OPL however remained tdTomato-, indicating that myeloid cells derived from infiltrating monocytes represented a relatively stable population. (**B**) High magnification view of the inset in (A) showing that monocyte-derived cells (Iba1+, tdTomato-; *white arrow*) taking up residence in the IPL developed mature ramified morphologies that were similar to those of endogenous microglia (Iba1+, tdTomato+; *yellow arrow*). Scale bar = 60 µm. Quantification of four separate morphological parameters (total dendrite length per cell, total number of dendrite segments per cell, total number of branch points per cell, and total cellular volume) of microglia in the IPL demonstrated no significant differences between monocyte-derived cells (Iba1+, tdTomato-; green bars) and endogenous microglia (Iba1+, tdTomato+; green-orange bars)($p > 0.05$ for all comparisons, unpaired t-test, n = 9 microglia in each group, 3 biological repeats from each group).

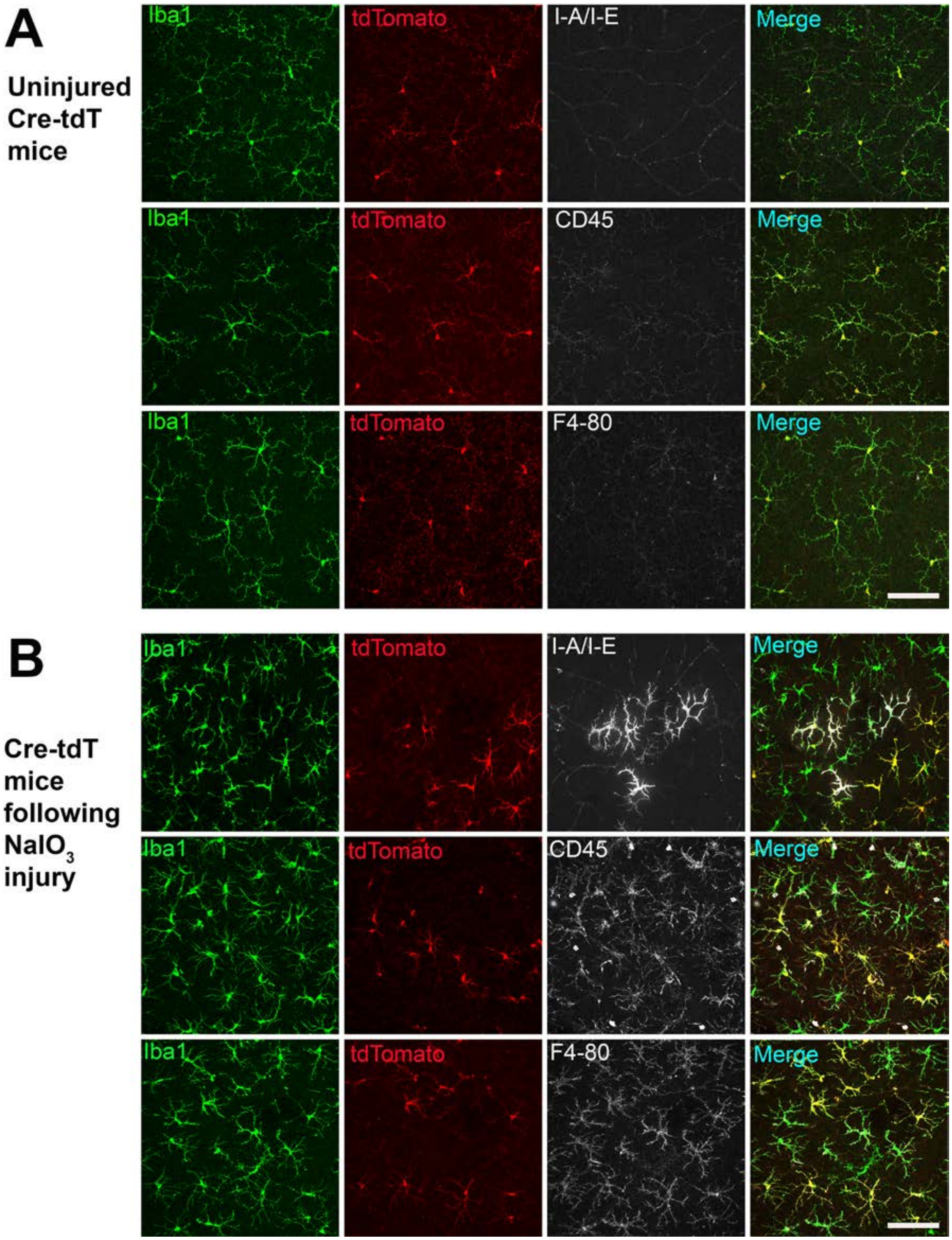
A
Uninjured
Cre-tdT
mice
Iba1
tdTomato
I-A/I-E
Merge
CD45
F4-80
B
Cre-tdT
mice
following
NaIO3
injury

Supplementary Figure 2. Myeloid cells originating from both infiltrating monocytes and endogenous microglia following $NaIO_3$-mediated RPE injury demonstrate increased expression of activation markers MHCII alloantigen I-A/I-E, CD45, and F4/80. (**A**) Three months following tamoxifen-mediated Cre recombination to "fate-map" endogenous retinal microglia with tdTomato, Cre-tdT transgenic mice were analyzed in the absence of retinal injury for immunopositivity for MHCII alloantigen I-A/I-E, CD45, and F4/80 in retinal flat-mounts. In the uninjured state, all Iba1+ myeloid cells in the inner retina were tdTomato+, indicating their status as endogenous microglia. These cells demonstrated very low or no immunopositivity to all three antigens. (**B**) Experimental mice that had been similarly "mapped" were subjected to $NaIO_3$ injury and then analyzed 3 months following injury. Inner retina myeloid cells comprised of both tdTomato+ microglia and tdTomato- cells that had originated from infiltrating monocytes. Immunopositivity for MHCII alloantigen I-A/I-E, CD45, and F4/80 were detectable in these myeloid cells, and can be found in both tdTomato+ and tdTomato- myeloid cells. Scale bar = 60 µm.

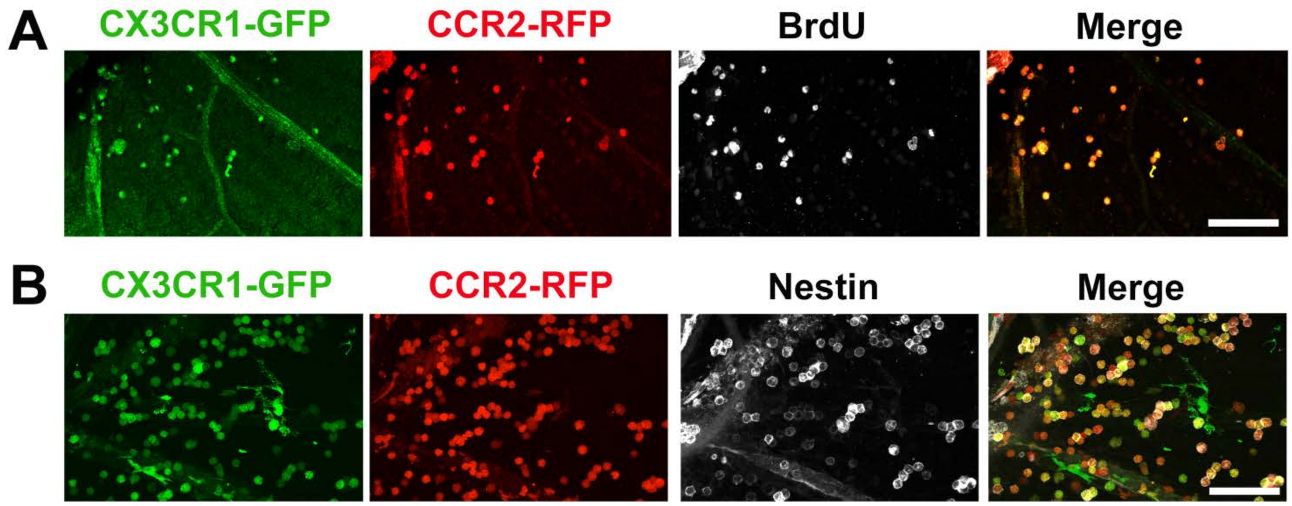

Supplementary Figure 3. CCR2-expressing monocytes infiltrating the retina demonstrate proliferation and nestin immunopositivity on entry into the GCL. $CX3CR1^{GFP/+}$:$CCR2^{RFP/+}$ transgenic mice subjected $NaIO_3$-mediated RPE injury were concurrently injected with BrdU (IP injection, 50μg/g body weight) and analyzed 2 days later with immunohistochemistry to BrdU and nestin. Imaging in the GCL of retinal flat-mounts revealed that the majority of CCR2-RFP+ infiltrating monocytes in the GCL were BrdU-immunopositive, indicating that they demonstrated prominent proliferation (*top row*). Nestin-immunopositivity in the GCL was observed in the majority of infiltrating monocytes (CX3CR1-GFP+, CCR2-RFP+) in the GCL, but not in endogenous microglia (CX3CR1-GFP+, CCR2-RFP-), indicating nestin expression to be a transient marker of infiltrating monocytes (*bottom row*). Scale bar = 60 µm.

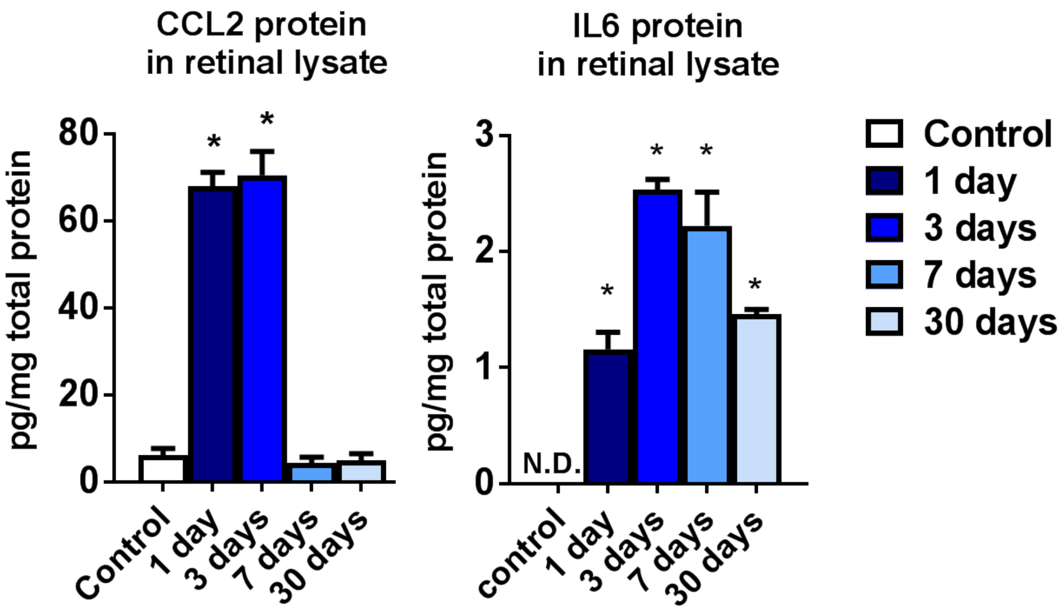

Supplementary Figure 4. Quantification of protein levels of CCL2 and IL6 in retinal lysates from $NaIO_3$-injured CX3CR1$^{GFP/+}$:CCR2$^{RFP/+}$ mice at various times following injury. CCL2 and IL6 levels increased markedly 1 day following injury and declined at time points >7 days after injury. (* indicates $p<0.05$ for comparisons relative to control, 1-way ANOVA with Sidak's multiple comparisons test, n = 3-4 independent replicates per group.)

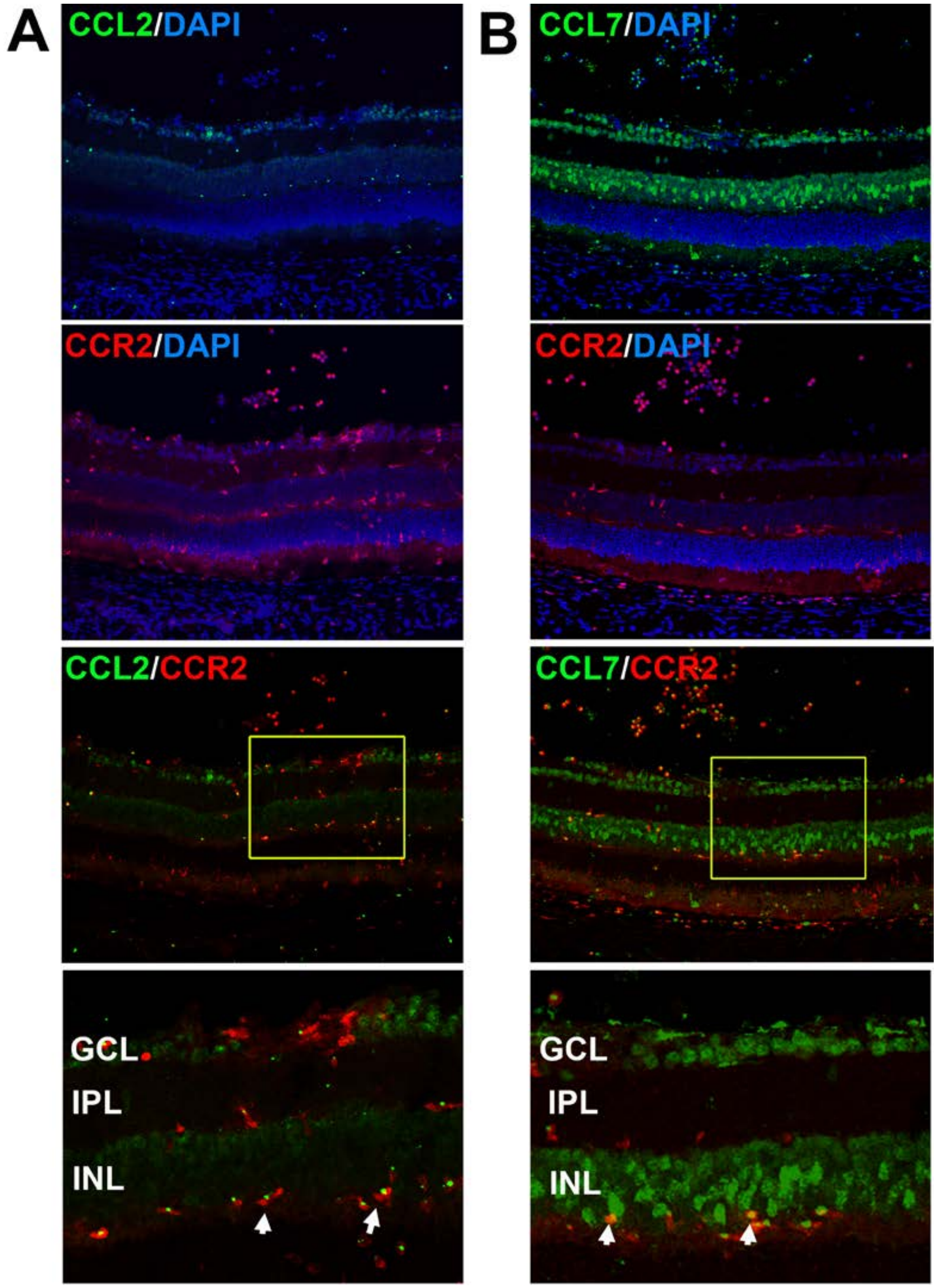

Supplementary Figure 5. Immunohistochemical localization of CCL2 and CCL7 expression following $NaIO_3$-mediated RPE injury. Immunohistochemistry to CCL2 (**A**) CCL7 (**B**) were performed in retinal sections from $CX3CR1^{GFP/}$;$CCR2^{RFP/+}$ mice 3 days followin g $NaIO_3$-mediated RPE injury. Insets show magnified fields outlined in boxes. CCL2 was expressed by CCR2+ infiltrating monocytes (*arrows*) as well as a subset of cells in the GCL. CCL7 was expressed by CCR2+ infiltrating monocytes (*arrows*) as well as cells in the GCL and INL. Scale bar = 200 µm.

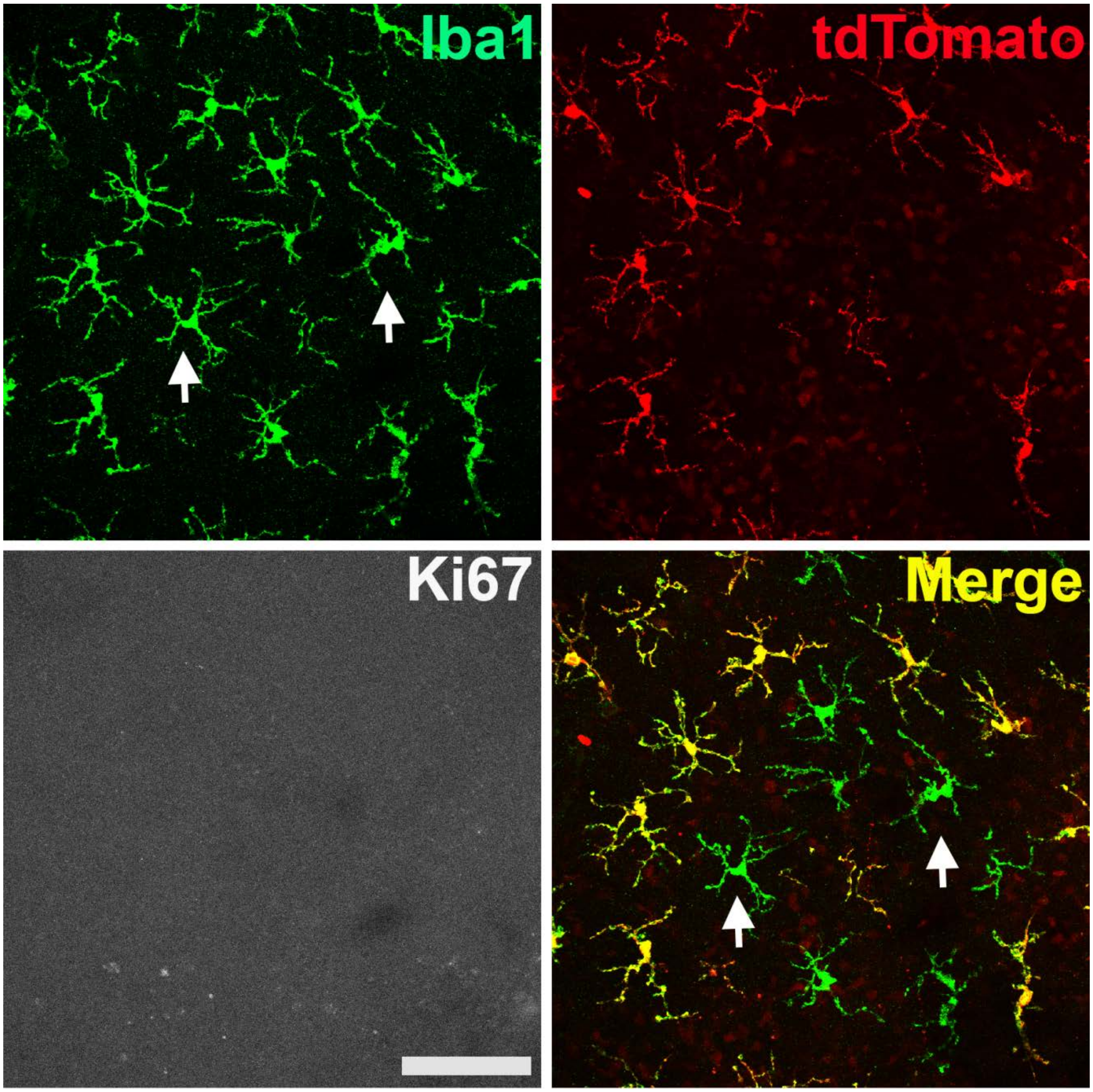

Supplementary Figure 6. Infiltrating monocytes recruited to the inner retina following $NaIO_3$-mediated RPE injury are persistent for up to at least 6 months following injury. Three months following tamoxifen-mediated Cre recombination to “fate-map” endogenous retinal microglia with tdTomato, Cre-tdT transgenic mice were subjected to $NaIO_3$-mediated RPE injury, and their retinas were analyzed 6 months following injury. Monocytes (Iba1+, tdTomato- cells) that have infiltrated the inner retina near the time of injury were still detectable 6 months following injury *(arrows)*. These cells are incorporated into the regular mosaic of Iba1+ cells, which consists of both tdTomato+ and tdTomato- cells. Staining for Ki67 also showed minimum immunopositivity, indicating infiltrated monocytes as a fairly stable population of long-lived cells in the retina. Scale bar = 60 µm.

Supplementary Table 1.

Gene	Forward Primer	Reverse Primer	Tm(°C)
GAPDH	CCTCTGGAAAGCTGTGGCG	CCTGCTTCACCACCTTCTTG	56
RPS13	CCTCCCCGAGGATCTCTAC	GAGCAGAGGCTGTGGATGAC	56
IL6	GAGTCCTTCAGAGAGATACAG	CTAGGTTTGCCGAGTAGATC	56
IL1β	GCTGGAGAGTGTGGATCCC	GACACGGATTCCATGGTGAAG	56
TNFα	CCAGGAGAAAGTCAACCTCC	GAGCAATGACTCCAAAGTAGAC	56
CCL2	CCTGGATCGGAACCAAATGA	CTAGTTCACTGTCACACTGG	56
CCL7	CATCCACATGCTGCTATGTC	CAAGGCTTTGGAGTTGGGG	56
CCL8	CACCTGCTGCTTTCATGTAC	CAAGGCTGCAGAATTTGAGAC	56
CCL12	CACGTCCGGAAGCTGAATAG	CACGTCCGGAAGCTGAATAG	56
